# SDD-PCN: Structure-Aware Dual-Decoder Learning for Joint Completion and Semantic Segmentation of Partial Indoor Point Clouds

**DOI:** 10.3390/s26196005

**Published:** 2026-09-22

**Authors:** Xiangrong Ni, Hanqiang Deng, Hao Chen, Sihang Zhou, Jian Huang

**Affiliations:** 1 College of Intelligence Science and Technology, National University of Defense Technology, Changsha 410073, China; nixiangrong23@nudt.edu.cn (X.N.); denghanqiang16@nudt.edu.cn (H.D.); chenhao@nudt.edu.cn (H.C.); sihangjoe@gmail.com (S.Z.); 2 National Key Laboratory of Equipment State Sensing and Smart Support, National University of Defense Technology, Changsha 410073, China

**Keywords:** point cloud completion, indoor scene understanding, semantic segmentation, structure-aware feature extraction, dual-decoder network, LiDAR simulation

## Abstract

Partial indoor point clouds acquired by light detection and ranging (LiDAR) are frequently affected by occlusion, restricted viewpoints, and non-uniform sampling, making it difficult to recover complete scene geometry while preserving the semantics of architectural structures and interior objects. To address this challenge, we propose the Structure-Aware Dual-Decoder Point Completion Network (SDD-PCN) for joint point-cloud completion and semantic segmentation. SDD-PCN first separates architectural structures from interior objects and applies dedicated multi-resolution encoders to capture their distinct geometric characteristics. The resulting local features are fused with a global scene representation and processed by parallel completion and segmentation streams that exchange information during progressive decoding. We further construct Partial-S3DIS, a partial-scene extension of the Stanford Large-Scale 3D Indoor Spaces (S3DIS) dataset, containing 1632 partial–complete scene pairs generated from 272 annotated rooms through sensor-aware LiDAR simulation. Experiments show that SDD-PCN achieves L1 and squared L2 Chamfer distance (CD-L1 and CD-L2) values of 16.73 and 15.27, respectively, under the ×$10^{-3}$ reporting convention. Relative to ProtoFormer, the strongest overall baseline, SDD-PCN reduces CD-L1 by 3.96% and CD-L2 by 7.90%, while obtaining the best completion performance in eight of ten scene categories. The model also achieves an overall accuracy (OA) of 87.23%, a mean class accuracy (mAcc) of 74.33%, and a mean intersection over union (mIoU) of 63.62%, outperforming both task-matched semantic scene completion baselines across all three semantic metrics. Ablation and robustness analyses confirm that structure-aware encoding and dual-stream feature interaction improve geometric recovery, particularly under moderate and severe missingness. These results demonstrate that explicitly modeling indoor structural heterogeneity provides an effective basis for unified point-based scene completion and semantic understanding.

## 1. Introduction

Three-dimensional point clouds provide a direct representation of scene geometry and have become an important data modality for indoor mapping, robotic navigation, digital-twin construction, and embodied scene understanding. However, point clouds acquired by LiDAR or depth sensors are inevitably incomplete because of occlusion, limited viewpoints, surface reflectance, sensing range, and sampling sparsity. Missing measurements can fragment object surfaces and erase structural boundaries, thereby reducing the reliability of subsequent reconstruction and semantic analysis. Point cloud completion therefore aims to infer geometrically plausible missing regions from partial observations and constitutes an important component of robust 3D perception systems [1,2].

Recent learning-based completion methods have substantially improved shape recovery by exploiting local edges, multi-scale geometry, graph relationships, global priors, and transformer-based feature interactions. Edge-aware generation preserves sharp geometric structures [3], while orientation- and scale-sensitive encoding improves the recovery of overall shapes and fine details [4]. Graph-guided deformation and rank-based graph aggregation further use neighborhood relationships to refine incomplete observations [5,6]. Global guidance and transformer-enhanced point deformation have also been used to establish long-range geometric dependencies [7,8]. More recently, PointSea combined self-projected multi-view representations with self-structure augmentation for global-to-local completion [9], while symmetry-guided progressive refinement was introduced to preserve structural regularities during point generation [10]. Nevertheless, these methods are predominantly designed and evaluated at the object level, where each input normally contains a single foreground instance with a relatively regular scale and topology.

Completing an indoor scene is fundamentally more challenging. A room simultaneously contains large architectural structures, such as floors, walls, ceilings, beams, and columns, and smaller interior objects with diverse shapes and spatial distributions. Treating these heterogeneous components as a single undifferentiated point set can cause dominant planar structures to suppress the features of small objects, while geometrically similar local regions may remain semantically ambiguous. Semantic scene completion addresses part of this problem by jointly reasoning about occupancy and semantic labels [11]. Recent studies have explored memory-augmented re-completion of invisible regions [12], geometry- and semantic-guided prediction, and generative scene completion [2,13,14]. Developments published in 2026 have further introduced dual-path fusion of voxel and bird’s-eye-view representations for single-frame LiDAR semantic scene completion [15] and completion-assisted multi-feature fusion for semantic segmentation of sparse point clouds [16]. However, these approaches generally operate in voxel or projected spaces, depend on image observations, or focus on outdoor driving scenes and sparse-input segmentation. Directly recovering dense indoor point sets while simultaneously predicting point-level semantics therefore remains insufficiently explored.

To address these challenges, we propose the Structure-Aware Dual-Decoder Point Completion Network, termed SDD-PCN, for joint geometric completion and semantic segmentation of partial indoor scenes. The central idea is to explicitly account for the geometric heterogeneity between architectural structures and interior objects. Given a partial scene, the structure-aware feature extractor organizes the input into structure-oriented and object-oriented subsets and learns dedicated representations for their distinct geometric characteristics. The resulting features are fused with a global scene representation, allowing for the network to retain dominant room layouts without discarding object-level details. A dual-decoder design subsequently maps the shared representation into two complementary outputs: a dense completed point cloud and pointwise semantic predictions. Joint optimization enables semantic cues to regularize geometric recovery, while the completed geometry provides spatial context for semantic reasoning.

Because existing completion benchmarks are dominated by isolated objects or complete indoor scans, we further construct Partial-S3DIS, a paired benchmark for partial indoor scene completion. Starting from 272 annotated rooms in S3DIS, we reconstruct scene surfaces and simulate six LiDAR observations for each room from different sensor poses. This procedure produces 1632 partial–complete pairs while preserving the original indoor semantic categories. Compared with artificial point removal based on simple cropping or random masking, ray-based LiDAR simulation generates viewpoint-dependent occlusion patterns and non-uniform sampling densities that more closely resemble practical indoor scans.

Experiments on Partial-S3DIS demonstrate that SDD-PCN consistently improves indoor scene completion. Under the $\times 10^{-3}$ reporting convention, the proposed method obtains CD-L1 and CD-L2 values of 16.73 and 15.27, respectively. Relative to ProtoFormer, the strongest overall baseline, SDD-PCN reduces CD-L1 by 3.96% and CD-L2 by 7.90%. It also achieves the best completion result in eight of the ten evaluated scene categories. Among the task-matched semantic scene completion methods, SDD-PCN obtains the highest OA of 87.23%, mAcc of 74.33%, and mIoU of 63.62%. In addition, the improvements observed under moderate and severe missingness confirm that explicitly modeling scene structure enhances robustness when only limited geometric evidence is available.

The main contributions of this work are summarized as follows:

- We introduce SDD-PCN, a unified point-based framework that jointly performs dense indoor scene completion and semantic segmentation through a shared encoder and two task-specific decoders.
- We develop a structure-aware feature extraction strategy that separately models architectural structures and interior objects before feature fusion, reducing feature interference between large planar components and geometrically diverse object instances.
- We construct Partial-S3DIS, containing 1632 paired partial and complete indoor scenes generated through multi-view LiDAR simulation, and conduct comprehensive comparison, ablation, robustness, and efficiency experiments to validate the proposed method.

## 2. Related Work

### 2.1. Object-Level Point Cloud Completion

Object-level point cloud completion aims to recover the complete geometry of an isolated object from a partial observation. Earlier learning-based studies focused on extracting a compact shape representation and decoding it into a plausible complete structure, including weakly supervised formulations that reduced the dependence on paired partial–complete samples [17]. Subsequent methods improved local feature aggregation and point generation through adaptive neighborhoods, dynamic transformers, hierarchical point splitting, and multi-scale folding [18,19]. More recent studies have introduced contour-aware voxel prediction, morphology-guided decomposition, canonical-space alignment, and edge-guided generation to preserve thin structures and handle complex or arbitrarily posed objects [20,21,22,23]. Although these methods have substantially improved object-level reconstruction, they are generally trained on normalized single-object datasets and rely on relatively stable category-level shape priors. Their direct extension to complete indoor scenes is therefore difficult because room-scale scans contain multiple semantic categories, dominant architectural surfaces, substantial scale variation, and complex occlusion relationships.

### 2.2. Indoor Scene Completion

Indoor scene completion requires the recovery of both global room layout and local object geometry from incomplete scans. Classical approaches frequently imposed geometric priors by fitting planes or other primitives to missing regions, which provided stable reconstruction for regular structures but had limited flexibility for irregular objects [24,25]. Recent surface reconstruction research has combined learned global and local priors to improve robustness to noise, non-uniform sampling, and missing points [26,27]. Noise suppression is also a general inverse problem in structured data: recent biomedical approaches recover latent gene-expression signals from sparse measurements or suppress indirect correlations in inferred gene-regulatory networks through statistical regularization and inverse diffusion [28,29]. In 3D sensing, recent methods have introduced adaptive stopping, deterministic residual diffusion, and learned structural priors to remove point perturbations while preserving geometric details [30,31,32]. Nevertheless, denoising refines corrupted observed points, whereas scene completion must infer entirely unobserved geometry; the two tasks are therefore related but not interchangeable. For indoor environments, semantic and primitive information has also been used to reconstruct walls, floors, furniture, and other scene components, while data-driven indoor scene modeling has increasingly emphasized contextual relationships among objects and architectural elements [33,34]. Nevertheless, many of these approaches produce meshes, implicit surfaces, primitive assemblies, or scene layouts rather than a completed point set with pointwise semantic labels. They also tend to treat architectural structures and interior objects within a common representation, leaving the strong geometric distinction between these two components insufficiently exploited.

### 2.3. Joint Completion and Semantic Segmentation

Joint completion and semantic segmentation are commonly formulated as semantic scene completion, in which a model predicts both the geometry or occupancy of unobserved regions and their semantic categories. Earlier studies primarily refined voxel predictions through dense conditional random fields or combined two-dimensional depth features with three-dimensional context and position-aware supervision [35,36]. Later methods introduced anisotropic convolutions, planar attention, and transformer-based feature lifting to model direction-dependent geometry and long-range semantic context [37,38,39,40]. However, voxel-based representations introduce a substantial resolution–memory trade-off, and many corresponding methods require image or depth-map inputs rather than directly generating completed point sets.

Point-based semantic scene completion provides a more directly comparable formulation for the present study. CasFusionNet connects global point completion, semantic segmentation, and local geometric refinement through a multilevel cascaded architecture with dense feature fusion [41]. More recently, ProtoFormer encoded class information into learnable semantic prototypes and used these prototypes to guide parallel geometric and semantic prediction in a transformer-based decoder [42]. Both methods directly generate point coordinates and pointwise semantic labels and are therefore included as task-matched baselines in our experiments. In contrast to these approaches, SDD-PCN explicitly decomposes an indoor scene into architectural structures and interior objects before joint decoding, allowing for geometrically heterogeneous scene components to be represented by dedicated feature encoders.

Recent point-based semantic scene completion methods provide more direct task-level counterparts to the present work. CasFusionNet progressively integrates multi-resolution point features through cascaded dense feature fusion to jointly recover scene geometry and semantic information [41]. ProtoFormer further introduces prototype-guided transformer representations to improve the semantic consistency of completed point clouds [42]. Unlike voxel-based semantic scene completion systems, these methods operate directly on point representations and can therefore be evaluated under the same point-cloud completion and semantic prediction protocol as SDD-PCN.

## 3. Proposed Method

This section presents SDD-PCN, a structure-aware dual-decoder network for the joint completion and semantic segmentation of partial indoor point clouds. The method directly processes unordered three-dimensional Cartesian coordinates (XYZ) without using red–green–blue (RGB) values, surface normals, voxel grids, or mesh representations. SDD-PCN follows a two-stage design. The Structure-Aware Feature Extraction (SFE) module first uses a Coarse Segmentation Unit to separate architectural structures from interior objects and extract a scene-level descriptor. Two structure-specific Fine Encoders then learn multi-resolution representations from the separated point sets. Finally, a Dual-Decoder progressively predicts complete point coordinates and their semantic labels at four resolutions.

### 3.1. Problem Formulation

Throughout this paper, calligraphic capital letters denote point sets or class sets, bold lowercase letters denote vectors or point coordinates, bold uppercase letters denote matrices or feature maps, and plain italic letters denote scalars, semantic labels, indices, or mappings. Superscripts identify the status of a point set or a network resolution, whereas subscripts identify individual points, structural branches, semantic classes, or network levels. In particular, the superscripts *p* and *g* denote a partial observation and its complete ground truth, respectively, and serve as descriptive labels rather than exponents. A hat denotes a predicted quantity. The branch subscripts arch and obj denote architectural structures and interior objects, respectively, while the superscripts *j* and *l* denote decoding and encoding levels when they appear in the subsequent formulation.

Let the partial point cloud of an indoor scene be denoted by

(1) $$P^{p}={\left\{x_{i}\in R^{3}\right\}}_{i=1}^{N_{p}},$$

where $x_{i}=\left(x_{i},y_{i},z_{i}\right)$ represents the three-dimensional coordinates of the *i*th observed point and $N_{p}$ is the number of partial-input points. The corresponding complete scene is represented as

(2) $$P^{g}={\left\{\left(q_{m},y_{m}\right)\right\}}_{m=1}^{N_{g}},$$

where $q_{m}\in R^{3}$ denotes a complete-scene point, $y_{m}\in \left\{1,\ldots ,C\right\}$ is its semantic label, $N_{g}$ is the number of ground-truth points, and C=13 is the number of semantic classes in S3DIS.

The semantic classes are further grouped into two coarse structural categories. The architectural category is defined as

(3) $$C_{\mathrm{arch}}=\left\{\mathrm{ceiling},\;\mathrm{floor},\;\mathrm{wall},\;\mathrm{beam},\;\mathrm{column},\;\mathrm{window},\;\mathrm{door}\right\},$$

whereas the interior-object category is defined as

(4) $$C_{\mathrm{obj}}=\left\{\mathrm{table},\;\mathrm{chair},\;\mathrm{sofa},\;\mathrm{bookcase},\;\mathrm{board},\;\mathrm{clutter}\right\}.$$

Define b(·):{1,…,C}→{0,1} as the binary structural-category mapping from a semantic label to its corresponding coarse category:

(5) $$b\left(y_{m}\right)=\left\{\begin{array}{cc} 0, & y_{m}\in C_{\mathrm{arch}},\\ 1, & y_{m}\in C_{\mathrm{obj}}. \end{array}\right.$$

Thus, $b(y_{m})=0$ identifies an architectural-structure point, whereas $b(y_{m})=1$ identifies an interior-object point.

Given $P^{p}$, the objective of SDD-PCN is to predict a complete semantic point cloud

(6) $$\hat{P}={\left\{\left({\hat{q}}_{k},{\hat{p}}_{k}\right)\right\}}_{k=1}^{M},$$

where ${\hat{q}}_{k}\in R^{3}$ is the predicted coordinate, ${\hat{p}}_{k}\in {\left[0,1\right]}^{C}$ is the corresponding semantic probability vector, and M=16,384 is the final output resolution. The predicted semantic label is obtained as

(7) $${\hat{y}}_{k}=\arg\max_{c\in \{1,\ldots ,C\}}{\hat{p}}_{k,c}.$$

Point-cloud completion does not provide a predefined one-to-one correspondence between generated and ground-truth points. SDD-PCN therefore learns geometric completion through a bidirectional set distance and obtains semantic supervision by matching each generated point to its nearest ground-truth point. This formulation allows for the network to optimize point coordinates and semantic labels within a unified multi-task framework.

Throughout this paper, calligraphic uppercase letters denote point sets, bold lowercase letters denote vectors, bold uppercase letters denote matrices or feature maps, and plain italic letters denote scalar values or indices. Superscripts *p* and *g* identify partial-input and ground-truth quantities, respectively, and are descriptive labels rather than exponents. A hat denotes a predicted quantity. Subscripts arch and obj identify the architectural and interior-object branches. The index *l* is reserved for Fine Encoder resolutions, whereas *j* denotes Dual-Decoder levels. The mapping b(·) converts a 13-class semantic label into its binary structural category. Variables used only in an individual equation are defined immediately after their first appearance. Table 1 summarizes the principal symbols used in the network forward pass and training objectives.

The notation table lists the symbols repeatedly used across the method. Neighborhood indices, sensor-ray parameters, metric-specific quantities, and other variables occurring only locally are additionally defined adjacent to their corresponding equations.

### 3.2. Overall Architecture

Figure 1 presents the forward pass of SDD-PCN as four sequential stages.

Figure 1a shows the coarse structural decomposition stage. Given the partial point set $P^{p}$, the Coarse Segmentation Unit predicts a binary structural label for every observed point and extracts the global scene feature $f_{g}$. The predicted labels divide the input into the architectural subset $P_{\mathrm{arch}}^{p}$ and the interior-object subset $P_{\mathrm{obj}}^{p}$.

Figure 1b shows the branch-specific encoding stage. The two subsets are processed by independently parameterized Fine Encoders. Each encoder aggregates local neighborhoods at four resolutions containing 128, 256, 512, and 2048 anchors, allowing for the architectural branch to model large planar relationships and the object branch to retain smaller and more irregular local structures.

Figure 1c illustrates global–local feature fusion. The 1024-dimensional global scene feature is projected to 128 dimensions, repeated across the final anchor set, and concatenated with the corresponding branch-specific local features. A 1×1 convolution transforms each concatenated representation into the initial fused feature $F_{r}^{0}$.

Figure 1d presents progressive dual decoding. Four Dual-Decoder Blocks increase the total output resolution from 512 to 2048, then to 8192, and finally to 16,384 points. Within each block, completion and semantic segmentation streams learn task-specific features and exchange information through the fused representation propagated to the next level. The final architectural and object predictions are concatenated along the point dimension to produce the completed coordinate matrix and semantic-probability matrix.

The Coarse Segmentation Unit adopts a PointNet++ encoder–decoder architecture [43]. It produces a binary structural probability for every input point and a 1024-dimensional global scene feature $f_{g}$. Based on the predicted binary labels, the partial input is divided into

(8) $$\begin{array}{cc} P_{\mathrm{arch}}^{p}\; & =\left\{x_{i}\in P^{p}:{\hat{b}}_{i}=0\right\}, \end{array}$$

(9) $$\begin{array}{cc} P_{\mathrm{obj}}^{p}\; & =\left\{x_{i}\in P^{p}:{\hat{b}}_{i}=1\right\}. \end{array}$$

The architectural branch processes ceilings, floors, walls, doors, windows, beams, and columns, whereas the object branch processes furniture, boards, and clutter.

Each partition is passed through its own Fine Encoder. The Fine Encoder extracts local features at four spatial resolutions and combines them with the global scene descriptor. The resulting branch-specific features are denoted by

(10) $$F_{r}^{0}=E_{r}\left(P_{r}^{p},f_{g}\right),\;r\in \left\{\mathrm{arch},\mathrm{obj}\right\},$$

where $E_{r}\left(\cdot \right)$ represents the corresponding Fine Encoder.

The two fused feature sets are subsequently processed by the branch-specific Dual-Decoders. Each Dual-Decoder contains a completion stream and a semantic segmentation stream. The two streams receive the same fused features, learn task-specific representations, and exchange information through the fused representation passed to the next decoding block. Four decoding blocks progressively increase the total output resolution according to

(11) $$M_{1}=512,\;M_{2}=2048,\;M_{3}=8192,\;M_{4}=16,384.$$

At each resolution, the output point budget is distributed between the architectural and object branches according to the structural proportions estimated by the Coarse Segmentation Unit:

(12) $$\begin{array}{cc} M_{\mathrm{arch}}^{j} & =\left\lfloor M_{j}\frac{\left|P_{\mathrm{arch}}^{p}\right|}{\left|P_{\mathrm{arch}}^{p}\right|+\left|P_{\mathrm{obj}}^{p}\right|}+0.5\right\rfloor , \end{array}$$

(13) $$\begin{array}{cc} M_{\mathrm{obj}}^{j} & =M_{j}-M_{\mathrm{arch}}^{j}. \end{array}$$

The two branch predictions are concatenated at each decoding level. The final output is therefore

(14) $$\hat{P}={\hat{P}}_{\mathrm{arch}}^{4}\cup {\hat{P}}_{\mathrm{obj}}^{4},\;\left|\hat{P}\right|=16,384.$$

Training is conducted in two stages. The Coarse Segmentation Unit is first trained for binary structural segmentation. Its parameters are subsequently frozen, and its predicted partitions and global feature are used to train the Fine Encoders and Dual-Decoders. The same predicted partitions are used during training and inference, preventing a discrepancy between the two stages.

### 3.3. Structure-Aware Feature Extraction

Indoor scenes contain two geometrically different forms of structure. Architectural elements are dominated by large planar surfaces, perpendicular boundaries, openings, and long-range spatial relationships. Interior objects contain smaller, denser, and more irregular local structures. Extracting a single representation from the entire scene can allow for the high-density architectural surfaces to dominate the features of smaller objects. The Structure-Aware Feature Extraction module addresses this problem by encoding the two structural partitions separately while preserving a shared global description of the complete scene.

#### 3.3.1. Coarse Structural Segmentation

The Coarse Segmentation Unit follows the hierarchical set-abstraction and feature-propagation design of PointNet++ [43]. Farthest point sampling is used to select representative centroids, and local neighborhoods are constructed around the centroids. Shared multilayer perceptrons and symmetric max pooling extract local geometric features, while feature-propagation layers restore per-point resolution. A two-channel classification head produces the architectural and object probabilities of every input point. The internal organization of the Structure-Aware Feature Extraction module is illustrated in Figure 2. The Coarse Segmentation Unit produces a global scene descriptor and assigns the observed points to the architectural-structure and interior-object subsets. Each subset is subsequently processed by a Fine Encoder that aggregates neighborhood information at multiple resolutions. The resulting local descriptors are combined with the global feature to form the fused representation used by the downstream decoders.

As detailed in Figure 2, the Fine Encoder progressively increases the spatial coverage of the extracted features while retaining local geometric evidence. At each resolution, farthest point sampling selects representative skeleton points, and *k*-nearest-neighbor grouping constructs their local neighborhoods. Features propagated from the preceding resolution are aggregated with the features extracted at the current resolution, allowing for the encoder to represent both local boundaries and larger structural patterns. Figure 2 also highlights that the two Fine Encoders have the same multi-resolution topology but do not share parameters. This distinction allows for the architectural branch to prioritize planar continuity, perpendicular boundaries, and long-range layout, while the object branch can represent smaller, denser, and more irregular neighborhoods. The global scene feature supplied to both branches preserves their common room-level context and prevents the structural decomposition from reducing the scene to two unrelated local representations. The deepest set-abstraction feature is aggregated through global max pooling to obtain

(15) $$f_{g}=\mathrm{MaxPool}\left(H_{g}\right)\in R^{1024},$$

where $H_{g}$ denotes the deepest pointwise feature map. Unlike the binary predictions, which determine the two structural partitions, $f_{g}$ summarizes the layout of the entire room and is supplied to both Fine Encoders. Consequently, each branch retains information about the spatial context in which its local structures occur.

#### 3.3.2. Multi-Resolution Local Encoding

For each structural branch r∈{arch,obj}, four anchor sets are generated through farthest point sampling:

(16) $$A_{1}=128,\;A_{2}=256,\;A_{3}=512,\;A_{4}=2048.$$

At resolution *l*, the anchor set is written as

(17) $$A_{r}^{l}={\left\{a_{r,u}^{l}\right\}}_{u=1}^{A_{l}}.$$

For every anchor point, the 16 nearest points in the corresponding structural partition are selected using Euclidean distance. The relative coordinate of a neighboring point is

(18) $$\Delta x_{r,u,k}^{l}=x_{r,u,k}^{l}-a_{r,u}^{l},\;k=1,\ldots ,16.$$

A shared multilayer perceptron with channel dimensions 3–64–128 maps each relative coordinate to a local feature. Symmetric max pooling then produces a permutation-invariant 128-dimensional descriptor:

(19) $$e_{r,u}^{l}=\max_{k=1,\ldots ,16}\phi_{l}\left(\Delta x_{r,u,k}^{l}\right),$$

where $\phi_{l}\left(\cdot \right)$ denotes the shared multilayer perceptron at resolution *l*. This operation follows the principle of learning directly from unordered point sets [44].

Features are propagated from the coarser anchor set to the next, denser anchor set. For each anchor $a_{r,u}^{l}$ with l>1, the three nearest anchors in $A_{r}^{l-1}$ are identified. Their features are aggregated using element-wise max pooling:

(20) $${\tilde{h}}_{r,u}^{l-1}=\max_{v\in N_{3}\left(a_{r,u}^{l},A_{r}^{l-1}\right)}h_{r,v}^{l-1}.$$

The propagated feature and the local feature extracted at the current resolution are concatenated and projected back to 128 dimensions:

(21) $$h_{r,u}^{l}=\psi_{l}\left(\left[e_{r,u}^{l}∥{\tilde{h}}_{r,u}^{l-1}\right]\right),$$

where ∥ denotes channel-wise concatenation and $\psi_{l}\left(\cdot \right)$ is a 1×1 convolution followed by batch normalization and a ReLU activation. At the first resolution,

(22) $$h_{r,u}^{1}=e_{r,u}^{1}.$$

This progressive feature propagation enables the encoder to combine sparse structural context with increasingly dense local details.

#### 3.3.3. Global–Local Feature Fusion

The 1024-dimensional global feature is projected to 128 dimensions:

(23) $${\tilde{f}}_{g}=\phi_{g}\left(f_{g}\right)\in R^{128}.$$

It is then replicated across the 2048 anchors of each structural branch and concatenated with the final local feature:

(24) $$F_{r}^{0}=\phi_{f}\left(\left[H_{r}^{4}∥\mathrm{Repeat}\left({\tilde{f}}_{g},2048\right)\right]\right)\in R^{2048\times 128},$$

where $H_{r}^{4}$ contains the local features at the fourth resolution and $\phi_{f}\left(\cdot \right)$ is a 1×1 convolution that reduces the concatenated representation to 128 channels. The fused feature $F_{r}^{0}$ simultaneously contains branch-specific local geometry and complete-scene contextual information.

### 3.4. Dual-Decoder

The Dual-Decoder performs coarse-to-fine point generation following the progressive decoding principle used in point-cloud completion networks [45]. Unlike a conventional single-task decoder, each Dual-Decoder Block (DD-Block) in SDD-PCN contains a completion stream and a semantic segmentation stream. These streams learn task-specific features but exchange information through the fused representation supplied to the next DD-Block. The internal architecture of the Dual-Decoder is illustrated in Figure 3. Each DD-Block contains parallel completion and semantic segmentation streams, and their task-specific features are fused before being transmitted to the next decoding level.

Figure 3 shows that completion and semantic prediction remain task-specific within each DD-Block but are not isolated. The completion stream provides geometric evidence about surface location and point displacement, whereas the semantic stream provides class-discriminative context. Their outputs are fused after every decoding level rather than only at the final output, enabling both forms of information to influence the subsequent, higher-resolution prediction. For structural branch *r* and decoding level *j*, the input feature is denoted by $F_{r}^{j-1}$. A feature-expansion operator first increases its spatial resolution to $M_{r}^{j}$. The expanded feature is independently processed by the completion and segmentation decoders:

(25) $$\begin{array}{cc} H_{r,\mathrm{com}}^{j} & =D_{\mathrm{com}}^{j}\left({\mathrm{Expand}}_{j}\left(F_{r}^{j-1}\right)\right), \end{array}$$

(26) $$\begin{array}{cc} H_{r,\mathrm{seg}}^{j} & =D_{\mathrm{seg}}^{j}\left({\mathrm{Expand}}_{j}\left(F_{r}^{j-1}\right)\right). \end{array}$$

Both decoders consist of two 1×1 convolutional layers, each followed by batch normalization and ReLU activation.

At the first decoding level, the completion stream directly predicts a coarse point skeleton:

(27) $${\hat{X}}_{r}^{1}={\mathrm{MLP}}_{\mathrm{xyz}}^{1}\left(H_{r,\mathrm{com}}^{1}\right)\in R^{M_{r}^{1}\times 3}.$$

At subsequent levels, the point set from the preceding level is duplicated according to the resolution-expansion ratio, and the completion stream predicts residual coordinate offsets:

(28) $${\hat{X}}_{r}^{j}=\mathrm{Up}\left({\hat{X}}_{r}^{j-1}\right)+\Delta {\hat{X}}_{r}^{j},\;j=2,3,4,$$

where

(29) $$\Delta {\hat{X}}_{r}^{j}={\mathrm{MLP}}_{\mathrm{offset}}^{j}\left(H_{r,\mathrm{com}}^{j}\right).$$

Residual coordinate prediction preserves the coarse structural layout while progressively refining surface details and object boundaries.

The segmentation stream predicts 13-dimensional semantic logits for every generated point:

(30) $$Z_{r}^{j}={\mathrm{MLP}}_{\mathrm{cls}}^{j}\left(H_{r,\mathrm{seg}}^{j}\right)\in R^{M_{r}^{j}\times C}.$$

The corresponding class probabilities are obtained through

(31) $${\hat{P}}_{r}^{j}=\mathrm{Softmax}\left(Z_{r}^{j}\right).$$

After the task-specific features have been extracted, they are concatenated with the expanded input feature and transformed into the fused representation for the next decoding level:

(32) $$F_{r}^{j}=\phi_{\mathrm{DD}}^{j}\left(\left[{\mathrm{Expand}}_{j}\left(F_{r}^{j-1}\right)∥H_{r,\mathrm{com}}^{j}∥H_{r,\mathrm{seg}}^{j}\right]\right),$$

where $\phi_{\mathrm{DD}}^{j}\left(\cdot \right)$ is a 1×1 convolution followed by batch normalization and ReLU activation. Consequently, geometric information extracted by the completion stream and contextual information extracted by the segmentation stream are both transmitted to the next decoding block. The interaction is repeated at all four resolutions, allowing for semantic and geometric information to influence progressively finer predictions.

At the final resolution, the branch-specific coordinate and semantic predictions are concatenated along the point dimension:

(33) $$\begin{array}{cc} \hat{X} & ={\mathrm{Concat}}_{0}\left({\hat{X}}_{\mathrm{arch}}^{4},{\hat{X}}_{\mathrm{obj}}^{4}\right), \end{array}$$

(34) $$\begin{array}{cc} \hat{P} & ={\mathrm{Concat}}_{0}\left({\hat{P}}_{\mathrm{arch}}^{4},{\hat{P}}_{\mathrm{obj}}^{4}\right), \end{array}$$

where ${\mathrm{Concat}}_{0}\left(\cdot ,\cdot \right)$ denotes row-wise concatenation along the point dimension.

### 3.5. Training Objectives

SDD-PCN is optimized in two stages. The first stage trains the Coarse Segmentation Unit. The second stage freezes this unit and jointly optimizes multi-resolution completion and semantic segmentation.

#### 3.5.1. Coarse Structural Segmentation Loss

For each partial-input point, let $b_{i}\in \left\{0,1\right\}$ denote the ground-truth structural label and let ${\hat{\pi }}_{i}\in \left[0,1\right]$ denote the predicted probability of belonging to the interior-object category. The binary structural segmentation loss is

(35) $$L_{\mathrm{coarse}}=-\frac{1}{N_{p}}\sum_{i=1}^{N_{p}}\left[b_{i}\log{\hat{\pi }}_{i}+\left(1-b_{i}\right)\log\left(1-{\hat{\pi }}_{i}\right)\right].$$

After convergence of the first-stage training, all parameters of the Coarse Segmentation Unit are frozen.

#### 3.5.2. Multi-Resolution Completion Loss

At decoding level *j*, the predicted point set for branch *r* is denoted by ${\hat{P}}_{r}^{j}$, and the corresponding complete ground-truth partition is denoted by $P_{r}^{g,j}$. The latter is obtained by farthest point sampling from the complete architectural or object partition at the resolution required by the corresponding decoding level.

The symmetric squared $L_{2}$ Chamfer distance between two point sets is defined as [46]

(36) $$\begin{array}{cc} {\mathrm{CD}}_{L2}\left({\hat{P}}_{r}^{j},P_{r}^{g,j}\right) & =\frac{1}{\left|{\hat{P}}_{r}^{j}\right|}\sum_{\hat{x}\in {\hat{P}}_{r}^{j}}\min_{q\in P_{r}^{g,j}}{∥\hat{x}-q∥}_{2}^{2}\\ \; & \;+\frac{1}{\left|P_{r}^{g,j}\right|}\sum_{q\in P_{r}^{g,j}}\min_{\hat{x}\in {\hat{P}}_{r}^{j}}{∥q-\hat{x}∥}_{2}^{2}. \end{array}$$

The completion loss at level *j* is the sum of the two structural branches:

(37) $$L_{\mathrm{com}}^{j}=\sum_{r\in \{\mathrm{arch},\mathrm{obj}\}}{\mathrm{CD}}_{L2}\left({\hat{P}}_{r}^{j},P_{r}^{g,j}\right).$$

The multi-resolution completion objective is

(38) $$L_{\mathrm{com}}=\sum_{j=1}^{4}\alpha_{j}L_{\mathrm{com}}^{j},$$

with

(39) $$\left(\alpha_{1},\alpha_{2},\alpha_{3},\alpha_{4}\right)=\left(0.1,0.2,0.5,1.0\right).$$

The increasing weights place stronger supervision on the final high-resolution prediction while preserving geometric constraints at the coarser levels.

#### 3.5.3. Multi-Resolution Semantic Segmentation Loss

Because generated points do not have predefined semantic correspondences, the target label of a predicted point is obtained from its nearest point in the complete ground-truth point cloud. For a generated point ${\hat{x}}_{r,i}^{j}$, its nearest ground-truth point index is

(40) $$\nu_{r,i}^{j}=\arg\min_{m}{∥{\hat{x}}_{r,i}^{j}-q_{r,m}^{g}∥}_{2},$$

and its semantic target is

(41) $$y_{r,i}^{j}=y_{r,\nu_{r,i}^{j}}^{g},$$

where $q_{r,m}^{g}$ and $y_{r,m}^{g}$ denote the coordinate and semantic label of the *m*th ground-truth point in branch *r*.

Let ${\hat{p}}_{r,i,c}^{j}$ denote the predicted probability that the point belongs to class *c*. The cross-entropy loss at decoding level *j* is

(42) $$L_{\mathrm{seg}}^{j}=-\sum_{r\in \{\mathrm{arch},\mathrm{obj}\}}\frac{1}{M_{r}^{j}}\sum_{i=1}^{M_{r}^{j}}\log{\hat{p}}_{r,i,y_{r,i}^{j}}^{j}.$$

The multi-resolution semantic objective is

(43) $$L_{\mathrm{seg}}=\sum_{j=1}^{4}\beta_{j}L_{\mathrm{seg}}^{j},$$

where

(44) $$\left(\beta_{1},\beta_{2},\beta_{3},\beta_{4}\right)=\left(0.1,0.2,0.5,1.0\right).$$

#### 3.5.4. Joint Objective

During the second training stage, the Fine Encoders and Dual-Decoders are jointly optimized using

(45) $$L_{\mathrm{joint}}=L_{\mathrm{com}}+\lambda_{\mathrm{seg}}L_{\mathrm{seg}},$$

where

(46) $$\lambda_{\mathrm{seg}}=0.1.$$

The completion loss remains the principal optimization objective, whereas semantic supervision regularizes the shared decoding features and encourages the generated points to follow semantically meaningful structures. Since the two streams exchange their task-specific representations after every decoding level, both losses contribute to the fused features used for subsequent point refinement.

## 4. Partial-S3DIS Dataset

Existing point-cloud completion datasets mainly focus on isolated objects and usually generate partial inputs through random point removal, fixed-view cropping, or artificial masking. Such procedures do not adequately reproduce the visibility constraints encountered during indoor LiDAR acquisition, where walls, furniture, sensor orientation, and platform motion jointly determine which surfaces can be observed. To provide paired partial and complete point clouds for room-level completion, we construct Partial-S3DIS from the Stanford Large-Scale 3D Indoor Spaces dataset (S3DIS) [47].

The construction pipeline contains three stages. First, the semantically annotated S3DIS point clouds are cleaned, downsampled, and converted into continuous triangular surfaces. Second, a configurable LiDAR simulator traces laser rays from different sensor trajectories and records the first visible surface intersection along each ray. Third, the resulting partial scans are paired with their corresponding complete point clouds and divided into non-overlapping training, validation, and test sets. The final dataset contains 272 complete rooms and 1632 simulated partial scans across ten indoor scene categories.

### 4.1. Source Data and Mesh Construction

#### 4.1.1. Source Point Clouds

S3DIS contains large-scale three-dimensional scans collected from six indoor areas and provides room-level point clouds with pointwise semantic annotations [47]. The original annotations follow the 13-class taxonomy

(47) $$C=\left\{\begin{array}{c} \mathrm{ceiling},\;\mathrm{floor},\;\mathrm{wall},\;\mathrm{beam},\;\mathrm{column},\;\mathrm{window},\;\mathrm{door},\\ \mathrm{table},\;\mathrm{chair},\;\mathrm{sofa},\;\mathrm{bookcase},\;\mathrm{board},\;\mathrm{clutter} \end{array}\right\}.$$

These labels are retained throughout Partial-S3DIS and serve as the ground truth for the semantic segmentation task described in Section 3.

The dataset construction uses all 272 room-level scenes in S3DIS. The rooms belong to ten functional categories: conference room, storage room, office, WC, lobby, lounge, auditorium, copy room, hallway, and pantry. Each room is processed independently so that its coordinate system, semantic annotations, and scene boundaries remain unchanged.

Before surface reconstruction, duplicated points and points with non-finite coordinates are removed. The remaining point cloud is voxel-downsampled using a voxel size of

(48) v=0.05m.

Within each occupied voxel, the retained coordinate is the centroid of the points in that voxel. Its semantic label is determined by majority voting among the original labels. Ties are resolved using the label of the point nearest to the voxel centroid. This operation reduces local sampling redundancy while preserving walls, furniture boundaries, door openings, and other structural details required for scene completion.

The downsampled S3DIS point cloud is retained as the complete target point cloud. The reconstructed mesh is used only as an intermediate visibility surface for LiDAR simulation. Consequently, geometric artifacts introduced during surface reconstruction do not replace or modify the original completion target.

#### 4.1.2. Normal Estimation and Orientation

Surface normals are estimated for every downsampled point through principal-component analysis of its 30 nearest neighbors. For point $x_{i}$, let

(49) $$N_{30}\left(x_{i}\right)={\left\{x_{i,k}\right\}}_{k=1}^{30}$$

denote its local neighborhood and let

(50) $${\bar{x}}_{i}=\frac{1}{30}\sum_{k=1}^{30}x_{i,k}$$

be the neighborhood centroid. The local covariance matrix is

(51) $$\Sigma_{i}=\frac{1}{30}\sum_{k=1}^{30}\left(x_{i,k}-{\bar{x}}_{i}\right){\left(x_{i,k}-{\bar{x}}_{i}\right)}^{T}.$$

The eigenvector associated with the smallest eigenvalue of $\Sigma_{i}$ is used as the estimated normal. Normal orientations are made locally consistent through minimum-spanning-tree propagation over the neighborhood graph.

#### 4.1.3. Triangular-Mesh Reconstruction

A continuous surface is required because direct subsampling of the original points cannot reproduce geometric occlusion between the sensor and unobserved surfaces. We therefore reconstruct a triangular mesh from each oriented point cloud using Screened Poisson Surface Reconstruction [48]. This method incorporates the input points as interpolation constraints and produces a continuous surface suitable for ray tracing.

The reconstruction uses an octree depth of 10, a reconstruction scale of 1.1, a minimum of 1.5 samples per octree node, and linear interpolation of the iso-surface vertices. The resulting mesh is cropped to the room bounding box expanded by 0.05 m in each direction. Degenerate triangles, duplicated vertices, duplicated faces, and non-manifold edges are removed. Disconnected components containing fewer than 500 triangles are also discarded because they primarily arise from isolated measurement noise.

Semantic information is transferred to the mesh after reconstruction. Each mesh vertex receives the semantic label of its nearest point in the downsampled S3DIS point cloud. A triangle label is then determined by majority voting among its three vertex labels. This semantic mesh allows for every simulated LiDAR return to inherit a valid class label while preserving the original 13-class annotation system.

### 4.2. LiDAR Simulation

#### 4.2.1. Sensor and Trajectory Configuration

The LiDAR simulator models visibility through explicit ray–triangle intersections. The sensor is positioned at a height of 1.20 m above the floor, representing a LiDAR mounted on a mobile indoor platform. For each room, the horizontal motion axis is aligned with the principal horizontal axis of the room footprint. The sensor trajectory passes through the horizontal room centroid and is clipped 0.50 m from the two room boundaries. Scanning poses are sampled at intervals of 0.25 m along this trajectory.

The sensor has a horizontal field of view of $90^{\circ }$ and a vertical field of view of $40^{\circ }$. The horizontal angular resolution is $0.2^{\circ }$, and the vertical angular resolution is $0.4^{\circ }$. The minimum and maximum sensing ranges are fixed at 0.30 m and 30.00 m, respectively. Only XYZ coordinates and semantic labels are retained; RGB values and intensity measurements are not included.

Let $o_{s}$ denote the sensor origin at pose *s*, and let $R_{s}$ represent the sensor rotation matrix. A ray corresponding to horizontal angle ϕ and vertical angle ψ is expressed as

(52) $$r_{s,\phi ,\psi }\left(t\right)=o_{s}+td_{s,\phi ,\psi },$$

where

(53) $$d_{s,\phi ,\psi }=R_{s}\left[\begin{array}{c} \cos\psi \cos\phi \\ \cos\psi \sin\phi \\ \sin\psi \end{array}\right].$$

Beam divergence is set to 0.3 mrad. Each nominal beam is represented by one central ray and four auxiliary rays uniformly distributed on the boundary of the divergence cone. The nearest valid return among the five rays is retained. Because S3DIS does not provide calibrated material reflectance, the simulation models geometric visibility and occlusion rather than intensity-dependent return loss.

#### 4.2.2. Ray Casting and Occlusion

For each emitted ray, intersections with the reconstructed triangular mesh are calculated using the Möller–Trumbore ray–triangle intersection algorithm [49]. Let T denote the set of mesh triangles. The retained distance is

(54) $$t^{*}=\min\left\{t:r_{s,\phi ,\psi }\left(t\right)\cap T\neq ⌀,\;T\in T,\;0.30\leq t\leq 30.00\right\}.$$

The simulated return is

(55) $$p_{s,\phi ,\psi }=o_{s}+t^{*}d_{s,\phi ,\psi }.$$

Only the nearest valid intersection is recorded. All surfaces located behind this intersection are excluded, thereby reproducing occlusion by walls, tables, cabinets, and other indoor objects. The semantic label of a simulated return is inherited from the intersected triangle. When a return lies on a boundary shared by triangles with different semantic labels, the label is selected from the triangle with the smallest positive intersection distance. The geometric principles of the simulator are illustrated in Figure 4. Surfaces visible to the LiDAR produce retained points, surfaces outside the field of view form sensor blind regions, and surfaces located behind the first intersection form object-occlusion regions.

Figure 4 illustrates how the first-return rule produces spatially correlated missing regions. A surface outside the field of view forms a sensor blind region, whereas a surface lying behind a nearer intersection forms an occlusion region. Because only the nearest valid intersection is retained, the resulting gaps follow the room layout, sensor direction, and object arrangement rather than independent random point removal.

#### 4.2.3. Six Partial-Scan Configurations

To generate systematic variation in scene incompleteness, three downward sensor pitch angles are combined with two motion directions. The pitch-angle set is

(56) $$\Theta =\left\{10^{\circ },20^{\circ },30^{\circ }\right\}.$$

For each angle, the platform traverses the trajectory in both the forward and backward directions. The sensor yaw is aligned with the direction of motion; consequently, reversing the trajectory rotates the viewing direction by $180^{\circ }$ and exposes a different set of surfaces.

The six scanning configurations are therefore

(57) S=Θ×{forward,backward}.

Each complete room produces one partial point cloud under each configuration. Figure 5 illustrates the six partial scans generated from the same complete indoor scene.

Figure 5 demonstrates that changing either pitch angle or motion direction alters the visible subset of the same room. Reversing the trajectory changes which wall and object surfaces face the sensor, while increasing the downward pitch redistributes sampling toward lower surfaces and changes the visibility of ceilings, walls, and elevated objects. The six scans therefore represent distinct acquisition geometries rather than repeated realizations of a single missing-point mask.

The returns collected from all poses along one trajectory are merged into a single partial point cloud. Repeated returns are removed through 0.05 m voxel downsampling, using the same voxel size as the complete target. Each retained partial point preserves the semantic label inherited from the intersected mesh triangle. Thus, every dataset sample contains:

- A partial XYZ point cloud.
- Pointwise semantic labels for the partial scan.
- The corresponding complete XYZ point cloud.
- Pointwise semantic labels for the complete scene.
- The sensor pitch angle and motion direction used to generate the partial scan.

### 4.3. Dataset Splits and Statistics

#### 4.3.1. Room-Level Data Partitioning

The train–validation–test division is performed at the room level before LiDAR simulation. All six partial scans generated from the same room are assigned to the same subset. This grouped splitting strategy prevents different simulated views of an identical room from appearing in both training and evaluation data.

Rooms are stratified by scene category using a fixed split seed of 2025. Categories containing only three rooms—lobby, lounge, auditorium, copy room, and pantry—contribute one room to each subset. The final division contains 179 training rooms, 47 validation rooms, and 46 test rooms. Because every room produces six partial scans, the corresponding numbers of partial–complete pairs are the following:

(58) $$N_{\mathrm{train}}=179\times 6=1074,$$

(59) $$N_{\mathrm{validation}}=47\times 6=282,$$

and

(60) $$N_{\mathrm{test}}=46\times 6=276.$$

The complete Partial-S3DIS dataset therefore contains

(61) $$N_{\mathrm{total}}=1074+282+276=1632$$

paired samples. The category-level room distribution is reported in Table 2.

Table 2 shows that offices and hallways account for most of the available rooms, whereas lobby, lounge, auditorium, copy-room, and pantry categories contain only three rooms each. The room-level split assigns all six scans from a given room to the same subset, preventing view-level leakage between training and testing. The rare categories contribute one room to each subset; their category-specific results should therefore be interpreted more cautiously than those for offices and hallways.

#### 4.3.2. Point Counts and Completion Ratio

After 0.05 m voxel downsampling, the complete room point clouds contain between $10^{4}$ and $10^{5}$ points, depending on room size and geometric complexity. The partial point count varies with scene layout, sensor pitch, platform direction, and the degree of object occlusion.

Let $N_{\mathrm{partial}}$ denote the number of points in a simulated partial scan and $N_{\mathrm{complete}}$ denote the number of points in its corresponding complete target. The completion ratio is defined as

(62) $$R_{\mathrm{comp}}=\frac{N_{\mathrm{partial}}}{N_{\mathrm{complete}}},\;0<R_{\mathrm{comp}}\leq 1.$$

A smaller value indicates that a larger proportion of the scene is missing and therefore represents a more difficult completion case.

Figure 6 presents the distribution of completion ratios across the ten scene categories. The distributions differ substantially because the same sensor configuration produces different visibility patterns in open rooms, narrow hallways, cluttered offices, storage rooms, and rooms containing large occluding objects. Lounge and hallway scenes generally retain a larger proportion of their complete points, whereas WC, storage, and copy-room scenes contain more severe occlusion cases. The within-category dispersion also confirms that the six sensor configurations produce multiple incompleteness levels rather than repeated versions of a single artificial mask.

Overall, Partial-S3DIS preserves the complete geometry and 13-class semantic annotations of S3DIS while introducing controlled, physically interpretable partial observations. The paired data support room-level point-cloud completion, semantic segmentation of generated points, multi-task learning, and robustness evaluation under different degrees of scene incompleteness.

## 5. Experiments

This section evaluates SDD-PCN on the Partial-S3DIS test set. The experiments address six questions: (1) whether SDD-PCN improves overall room-level completion; (2) whether the improvement remains consistent across different scene categories; (3) whether the jointly generated semantic labels remain competitive with labels produced by independent segmentation networks; (4) how much the Structure-Aware Feature Extraction module and Dual-Decoder contribute to completion accuracy; (5) whether the method remains robust under different degrees of incompleteness; and (6) whether the joint architecture avoids the additional inference stage required by a completion-plus-segmentation pipeline.

### 5.1. Implementation Details

#### 5.1.1. Data Preprocessing

The implementation uses XYZ coordinates without RGB values, intensity information, or surface normals. Before network input, each partial–complete pair is transformed using the same room-level normalization. Let c denote the center of the complete-scene bounding box and let $l_{\max}$ denote its longest side. Every partial and complete point is normalized as

(63) $$\tilde{x}=\frac{x-c}{l_{\max}}.$$

This transformation centers the room at the coordinate origin and makes the largest bounding-box dimension equal to one.

Each partial input is sampled to 16,384 points. Farthest point sampling is used when the partial scan contains more than 16,384 points. When it contains fewer points, all available points are retained and the remaining positions are filled through uniform resampling with replacement. Complete target point clouds are sampled to 16,384 points using farthest point sampling. The completion ratio defined in Equation (62) is always calculated before this fixed-size network sampling.

Training data are augmented through random rotations around the vertical axis, isotropic scaling in the range [0.90,1.10], and coordinate jitter sampled from a Gaussian distribution with standard deviation 0.005 and clipped to [−0.02,0.02]. The same transformation is applied to the partial input and complete target. Validation and test data are not augmented.

#### 5.1.2. Training Configuration

SDD-PCN is implemented in Python 3.9 and PyTorch 1.13.1 with CUDA 11.7. All experiments are conducted on a workstation equipped with an NVIDIA GeForce RTX 3090 GPU (NVIDIA Corporation, Santa Clara, CA, USA) with 24 GB of memory. The random seed is fixed at 2025 for data sampling, parameter initialization, and data augmentation. The Coarse Segmentation Unit is trained first. It is optimized for 100 epochs using Adam with an initial learning rate of $1\times 10^{-3}$, a weight decay of $1\times 10^{-4}$, and a batch size of 8. The learning rate follows cosine annealing and decreases to $1\times 10^{-5}$. The checkpoint with the lowest validation binary cross-entropy loss is retained. The minimum validation loss of 0.174 is obtained at epoch 88, at which the corresponding training loss is 0.162. This checkpoint is retained and its parameters are frozen before stage 2. The trained Coarse Segmentation Unit is then frozen. The two Fine Encoders and Dual-Decoders are jointly trained for 250 epochs using Adam with an initial learning rate of $1\times 10^{-4}$, a weight decay of $1\times 10^{-4}$, and a batch size of 4. The learning rate is reduced to $1\times 10^{-6}$ through cosine annealing. The multi-resolution loss weights and semantic-loss coefficient follow Equations (39), (44), and (46), respectively. The final checkpoint is selected according to the lowest validation CD-L2. The minimum validation CD-L2 of 15.64 under the $\times 10^{-3}$ reporting convention is obtained at epoch 218, and this checkpoint is used for all final test-set evaluations.

Figure 7a shows that the stage-1 training and validation binary cross-entropy losses decrease rapidly during the first 30 epochs and remain closely aligned throughout the 100-epoch optimization procedure. The training loss decreases from 0.684 to below 0.250 during the initial 30 epochs. The minimum validation loss of 0.174 is obtained at epoch 88, at which the training loss is 0.162. The absence of a sustained separation between the training and validation curves indicates stable convergence without marked overfitting during coarse structural segmentation.

As shown in Figure 7b, the stage-2 completion loss, weighted semantic loss, and joint loss decrease steadily and approach stable plateaus. The joint loss remains equal to the sum of the completion loss and the weighted semantic contribution, consistent with the specified multi-task objective. Neither component exhibits sustained oscillation or reversal, indicating stable joint optimization, although the loss trajectories alone cannot exclude all forms of task-level gradient conflict.

Figure 7c shows that validation CD-L2 decreases from 36.42 at epoch 1 to below 20.00 by epoch 65 and subsequently enters a slower refinement phase under cosine learning-rate annealing. The minimum validation CD-L2 of 15.64 is obtained at epoch 218, and this checkpoint is selected for the final test evaluation. After epoch 220, validation CD-L2 remains on a nearly flat plateau with fluctuations of approximately ±0.15 and shows no sustained upward trend. Therefore, no marked late-stage overfitting is observed under the applied optimization, multi-resolution supervision, weight decay, and data-augmentation settings.

#### 5.1.3. Comparison Protocol

Six representative networks are included as baselines. Four object-level point-cloud completion methods—PF-Net [50], GRNet [51], SnowflakeNet [52], and CRA-PCN [53]—represent hierarchical generation, grid-based completion, progressive point deconvolution, and cross-resolution transformer architectures, respectively. Two point-based semantic scene completion methods are additionally included. CasFusionNet [41] performs cascaded global completion, semantic segmentation, and local refinement through dense feature fusion, whereas ProtoFormer [42] uses learnable semantic prototypes to guide transformer-based geometric and semantic decoding.

All six baseline models were retrained from scratch on the same 1074 Partial-S3DIS training pairs and selected using the same 282 validation pairs. Their official network structures and recommended optimization settings were retained wherever applicable. Only the input layer, output resolution, and semantic classification head were adapted when necessary to process XYZ coordinates, generate 16,384 points, and predict the 13 Partial-S3DIS semantic classes. The final models were selected according to validation CD-L2, consistent with the selection criterion used for SDD-PCN.

All baselines received only the normalized XYZ coordinates used by SDD-PCN. No RGB information, ground-truth semantic labels, reconstructed mesh surfaces, or sensor-pose metadata were provided as model inputs. Geometric performance was evaluated for all methods, while semantic metrics were additionally evaluated for CasFusionNet, ProtoFormer, and SDD-PCN because these three methods jointly generate completed coordinates and pointwise semantic labels. All overall results were computed as strict pair-level averages over the same 276 test scenes.

### 5.2. Evaluation Metrics

#### 5.2.1. Point-Cloud Completion Metrics

Completion quality is evaluated using the bidirectional L1 and squared L2 Chamfer distances. Let $\hat{P}$ denote a predicted complete point cloud and $P^{g}$ denote its ground-truth complete point cloud. CD-L1 is defined as

(64) $$\begin{array}{cc} {\mathrm{CD}}_{L1}\left(\hat{P},P^{g}\right)\; & =\frac{1}{\left|\hat{P}\right|}\sum_{\hat{x}\in \hat{P}}\min_{q\in P^{g}}{∥\hat{x}-q∥}_{2}\\ \; & \;+\frac{1}{\left|P^{g}\right|}\sum_{q\in P^{g}}\min_{\hat{x}\in \hat{P}}{∥q-\hat{x}∥}_{2}, \end{array}$$

whereas CD-L2 uses the squared Euclidean distance defined in Equation (36), evaluated on the full predicted and ground-truth point sets.

Lower values indicate better geometric completion. Both metrics are multiplied by $10^{3}$ when reported in the tables.

#### 5.2.2. Structure-Specific Geometric Metrics

Chamfer distance measures overall geometric proximity but does not directly quantify the regularity of dominant planar structures or the generation of unsupported points around door and window regions. We therefore introduce plane fitting error (PFE) and a door/window-region false-filling ratio (FFR).

For each test scene *s* and planar semantic category c∈{ceiling,floor,wall}, the corresponding ground-truth points are decomposed into planar patches using RANSAC with a distance threshold of 0.05 m. Let $G_{s,c,k}$ denote the *k*th ground-truth plane patch and let its fitted plane be

(65) $$\Pi_{s,c,k}=\left\{x:n_{s,c,k}^{T}x+d_{s,c,k}=0\right\},\;{∥n_{s,c,k}∥}_{2}=1.$$

Each generated point is assigned to the plane patch containing its nearest ground-truth point. If ${\hat{P}}_{s,c,k}$ denotes the generated points assigned to $G_{s,c,k}$, the patch-level PFE is

(66) $${\mathrm{PFE}}_{s,c,k}=\frac{1}{\left|{\hat{P}}_{s,c,k}\right|}\sum_{\hat{x}\in {\hat{P}}_{s,c,k}}\left|n_{s,c,k}^{T}\hat{x}+d_{s,c,k}\right|.$$

The errors are calculated after transforming the point clouds back to metric coordinates. Patch-level errors are first averaged within each scene, followed by macro-averaging across test scenes. PFE is reported in centimeters, and a lower value indicates better preservation of planar geometry.

For c∈{door,window}, let $\Omega_{s,c}$ denote the union of the three-dimensional bounding boxes of the corresponding ground-truth instances, expanded by 0.05 m in each direction. A generated point inside $\Omega_{s,c}$ is considered falsely filled if its distance to the complete ground-truth point cloud exceeds τ=0.05 m. The scene-level FFR is defined as

(67) $${\mathrm{FFR}}_{s,c}=\frac{\sum_{\hat{x}\in {\hat{P}}_{s}}I\left[\hat{x}\in \Omega_{s,c}\right]I\left[\min_{q\in P_{s}^{g}}{∥\hat{x}-q∥}_{2}>\tau \right]}{\sum_{\hat{x}\in {\hat{P}}_{s}}I\left[\hat{x}\in \Omega_{s,c}\right]+\epsilon },$$

where I[·] is the indicator function and ϵ prevents division by zero. The ratios are macro-averaged over test scenes containing the corresponding ground-truth category and are reported as percentages. A lower FFR indicates fewer unsupported generated points in door or window regions.

#### 5.2.3. Semantic Segmentation Metrics

Because the output points are generated rather than copied from the ground truth, the reference semantic label of each predicted point is obtained from its nearest ground-truth point, as defined in Section 3.5. Semantic performance is evaluated using overall accuracy (OA), mean class accuracy (mAcc), and mean intersection over union (mIoU).

Overall accuracy is

(68) $$\mathrm{OA}=\frac{\sum_{c=1}^{C}{\mathrm{TP}}_{c}}{\sum_{c=1}^{C}\left({\mathrm{TP}}_{c}+{\mathrm{FN}}_{c}\right)}.$$

Mean class accuracy is

(69) $$\mathrm{mAcc}=\frac{1}{C}\sum_{c=1}^{C}\frac{{\mathrm{TP}}_{c}}{{\mathrm{TP}}_{c}+{\mathrm{FN}}_{c}},$$

and mean intersection over union is

(70) $$\mathrm{mIoU}=\frac{1}{C}\sum_{c=1}^{C}\frac{{\mathrm{TP}}_{c}}{{\mathrm{TP}}_{c}+{\mathrm{FP}}_{c}+{\mathrm{FN}}_{c}}.$$

All three semantic metrics are reported as percentages.

### 5.3. Comparison with Baselines

#### 5.3.1. Overall Completion Performance

Table 3 reports the overall geometric completion results on the 276 Partial-S3DIS test pairs. In addition to the four object-level completion baselines, the comparison now includes the point-based semantic scene completion methods CasFusionNet and ProtoFormer.

Both task-matched semantic scene completion baselines outperform the strongest object-level completion baseline. CasFusionNet reduces CD-L2 from the 18.95 obtained by CRA-PCN to 18.27, while ProtoFormer further reduces it to 16.58. SDD-PCN achieved CD-L1 and CD-L2 values of 16.73 and 15.27, respectively. Relative to ProtoFormer, the strongest overall baseline, SDD-PCN reduced CD-L1 by 3.96% and CD-L2 by 7.90%; relative to CRA-PCN, the strongest object-level completion baseline, the corresponding reductions were 12.91% and 19.42%. These results show that the advantage of SDD-PCN remains present when it is compared with methods that, like SDD-PCN, jointly learn geometric completion and semantic prediction.

#### 5.3.2. Category-Level Completion Performance

Table 4 presents CD-L2 separately for the ten scene categories. The overall pair-level averages are reported separately in Table 3 and are therefore not repeated as an unweighted category mean.

SDD-PCN obtains the lowest CD-L2 in eight of the ten scene categories and outperforms both CasFusionNet and ProtoFormer in every category. Its advantage over ProtoFormer is particularly apparent in hallways, storage rooms, WCs, conference rooms, and auditoriums, where the simultaneous recovery of large architectural surfaces, openings, and smaller interior structures is required. GRNet remains the best-performing method in the lobby and lounge categories, with CD-L2 values of 12.07 and 12.97, respectively; SDD-PCN ranks second in both categories. The expanded comparison therefore indicates that the category-level advantage of SDD-PCN persists against both object-level completion networks and task-matched semantic scene completion methods.

#### 5.3.3. Structure-Specific Geometric Evaluation

Table 5 evaluates planar regularity and false filling around door and window regions. These metrics complement Chamfer distance by focusing on geometrically important indoor structures.

SDD-PCN obtains the lowest ceiling and wall PFE and reduces mean PFE from 2.09 cm for ProtoFormer to 1.80 cm, corresponding to a reduction of 13.88%. Its door FFR of 12.3% is also 25.00% lower than the 16.4% obtained by ProtoFormer. These results indicate that structure-aware encoding improves the recovery of dominant indoor planes and suppresses unsupported generation around door regions.

The improvement is not universal across all local structures. ProtoFormer obtains a slightly lower floor PFE of 1.83 cm compared with 1.85 cm for SDD-PCN and a lower window FFR of 14.7% compared with 15.2%. The structure-specific metrics therefore support an overall improvement in architectural reconstruction while identifying the floor and window regions in which SDD-PCN does not achieve the best result.

#### 5.3.4. Qualitative Comparison

Figure 8 compares the geometric outputs of all methods. The same camera pose, point size, coordinate system, and height-based color mapping are used within each scene. Warmer colors represent larger vertical coordinates, whereas cooler colors represent points closer to the floor. In Figure 8 and Figure 9, GT denotes the corresponding ground-truth complete point cloud.

PF-Net frequently generates scattered points outside room boundaries and oversmooths large missing regions. GRNet better preserves global room volume but produces irregular ceiling and floor distributions in several categories. SnowflakeNet generates relatively dense surfaces but often fills openings that should remain empty. CRA-PCN reconstructs cleaner global structures, although visible distortions remain around room boundaries and cavities. SDD-PCN produces more regular ceiling–wall–floor relationships in conference rooms, offices, WCs, and hallways. In lobby and copy-room scenes, it preserves larger door and window openings and generates fewer isolated points outside the architectural boundaries. In lounge, pantry, and auditorium scenes, the vertical color distribution more closely follows the ground truth, indicating improved recovery of relative ceiling, floor, and object heights. The visual results are consistent with the lower category-level Chamfer distances reported in Table 4.

Figure 9 provides the corresponding qualitative ablation comparison, discussed in Section 5.5.

### 5.4. Semantic Segmentation Results

Because CasFusionNet, ProtoFormer, and SDD-PCN all jointly predict completed coordinates and pointwise semantic labels, Table 6 compares their geometric and semantic performance under the same Partial-S3DIS evaluation protocol.

SDD-PCN achieves the best result for all five joint-task metrics. Compared with ProtoFormer, it reduces CD-L1 and CD-L2 by 3.96% and 7.90%, respectively, while improving OA by 0.52 percentage points, mAcc by 0.68 percentage points, and mIoU by 1.44 percentage points. The improvement in both metric families indicates that the geometric advantage of SDD-PCN is not obtained at the expense of semantic prediction. Instead, the structure-aware branches and cross-task feature interaction support both completed-coordinate generation and semantic discrimination.

To place these joint-task results in the context of dedicated post-completion segmentation networks, we additionally retain the comparison with PointNet, PointCNN, SPGraph, PointWeb, and ShellNet below.

The semantic decoder of SDD-PCN predicts a 13-class label for every generated point. To determine whether this integrated semantic head remains competitive with a separate post-completion segmentation model, five established segmentation networks are evaluated: PointNet [44], PointCNN [54], SPGraph [55], PointWeb [56], and ShellNet [57].

The five independent segmentation networks are trained on the complete point clouds in the Partial-S3DIS training set. At test time, they receive the completed XYZ coordinates generated by SDD-PCN and predict semantic labels as a separate post-processing stage. SDD-PCN instead uses the labels generated by its own Dual-Decoder. Therefore, the comparison evaluates the integrated semantic decoder against independently trained segmentation networks under the same completed geometry.

Table 7 summarizes the semantic segmentation results. SDD-PCN achieves an OA of 87.23%, an mAcc of 74.33%, and an mIoU of 63.62%. It substantially outperforms PointNet and also exceeds SPGraph by 1.66 percentage points in OA, 1.31 points in mAcc, and 1.48 points in mIoU. PointCNN obtains the highest OA, PointWeb obtains the highest mAcc, and ShellNet obtains the highest mIoU.

The integrated decoder is therefore not the strongest stand-alone semantic segmenter. Its principal advantage is that it produces semantic labels during geometric completion without requiring a second network. The results show that the joint semantic branch retains competitive segmentation performance while simultaneously providing semantic features to the completion stream.

#### Per-Class Semantic Results

To examine whether the aggregate semantic improvements are distributed consistently across categories, Table 8 reports the IoU of all 13 Partial-S3DIS semantic classes for the three joint completion and semantic prediction methods.

SDD-PCN achieves the highest IoU in 10 of the 13 semantic classes, including five architectural classes and five interior-object classes. Compared with ProtoFormer, the largest improvements occur for beam, door, bookcase, and board. These results indicate that the overall mIoU improvement is not driven by only one or two dominant classes.

ProtoFormer obtains the highest IoU for floor and sofa, whereas CasFusionNet performs best for clutter. The relatively lower IoUs for beam, column, window, clutter, and several less frequent object classes across all three methods also indicate that small structures, class imbalance, and substantial occlusion remain challenging.

### 5.5. Ablation Study

The ablation study evaluates the incremental contributions of the Structure-Aware Feature Extraction module and the Dual-Decoder. Three configurations are compared:

1. Standard encoder–decoder: a single conventional point-cloud encoder and completion decoder.
2. Standard decoder with SFE: the conventional encoder is replaced by the proposed structure-aware coarse segmentation and Fine Encoders.
3. Complete SDD-PCN: the SFE module is combined with the Dual-Decoder and semantic supervision.

All configurations use the same training data, output resolution, optimizer, training schedule, and completion loss. The results are reported in Table 9.

Replacing the standard encoder with SFE reduces the overall pair-level CD-L2 from 27.94 to 19.01, corresponding to a 31.96% reduction. This improvement occurs in every scene category, supporting the separation of large architectural surfaces and smaller interior structures before detailed feature extraction. Adding the Dual-Decoder further reduces CD-L2 from 19.01 to 15.27, an additional reduction of 19.67%. Relative to the standard encoder–decoder, the complete SDD-PCN reduces the overall completion error by 45.35%. The consistent improvements across all ten categories indicate that semantic supervision and cross-task feature transmission provide useful constraints during progressive point generation.

Figure 9 visualizes the ablation process for a hallway scene. The standard encoder–decoder produces numerous outliers outside the ceiling, walls, and floor and incorrectly fills the central opening. SFE suppresses many of these errors and better preserves the room boundary. The complete SDD-PCN further removes scattered points and reconstructs the opening more accurately.

For this example, SFE reduces CD-L2 by 32.94%, whereas the Dual-Decoder produces a further reduction of 39.92% relative to the SFE-only configuration. The complete model reduces the error by 59.71% relative to the conventional encoder–decoder.

#### Shared Versus Branch-Specific Encoding

To separately examine the contributions of structural partitioning and branch-specific feature learning, we compare three encoder configurations under the same Dual-Decoder architecture. The unified shared encoder directly encodes the complete partial scene without separating architectural structures from interior objects. The partitioned shared-weight encoder retains the predicted structural partition but processes the two subsets using Fine Encoders with shared parameters. The branch-specific encoders correspond to the complete SDD-PCN design, in which the architectural and interior-object subsets are processed by independently parameterized Fine Encoders. All other network components, training data, optimization settings, output resolutions, and loss functions are kept unchanged.

As reported in Table 10, introducing structural partitioning while retaining shared encoder weights reduces CD-L1 from 21.14 to 18.67 and CD-L2 from 19.63 to 17.39, corresponding to reductions of 11.68% and 11.41%, respectively. It also improves OA, mAcc, and mIoU by 3.04, 3.34, and 5.91 percentage points. These improvements show that separating architectural structures from interior objects is beneficial even when the two partitions are processed using the same encoder parameters.

Replacing the shared-weight encoder with branch-specific encoders further reduces CD-L1 and CD-L2 by 10.39% and 12.19%, respectively, and improves OA, mAcc, and mIoU by 2.02, 2.47, and 3.87 percentage points. Relative to the unified shared-encoder configuration, the complete SDD-PCN reduces CD-L1 and CD-L2 by 20.86% and 22.21% and increases mIoU by 9.78 percentage points. The results distinguish the benefit of structural partitioning from the additional benefit of learning separate feature transformations for architectural structures and interior objects.

Figure 10 further compares the representations learned by the shared-weight and branch-specific encoders. In the shared-weight configuration, the architectural and interior-object samples substantially overlap in the t-SNE projection, indicating limited separation between the two structural feature distributions. In contrast, the branch-specific encoders produce a clear separation between the architectural and interior-object samples, with the interior-object features forming several compact local clusters. Consistent with this observation, the silhouette score calculated from the original 128-dimensional fused features increases from 0.204 for the shared-weight encoder to 0.461 for the branch-specific encoders. The semantic-class centroid heatmaps provide complementary evidence. When the encoder weights are shared, considerable feature similarity remains across the boundary between the seven architectural classes and the six interior-object classes. With branch-specific encoding, the cross-branch similarities become substantially lower, while coherent within-branch relationships are retained. These results indicate that the improvement does not arise solely from dividing the input point cloud. Independently parameterized encoders allow for the two branches to learn feature spaces adapted to the planar and large-scale geometry of architectural structures and the smaller, irregular geometry of interior objects.

### 5.6. Robustness and Efficiency

#### 5.6.1. Robustness to Input and Acquisition Perturbations

The completion-ratio analysis evaluates robustness to the amount of observed scene geometry but does not directly examine measurement errors, reduced sampling density, or deviations from the nominal sensor trajectory. We therefore conduct three additional test-time perturbation experiments without retraining the evaluated models.

For the noise experiment, zero-mean Gaussian range noise with standard deviations of 0.005, 0.010, and 0.020 m is added along the corresponding LiDAR ray direction before room-level normalization. For the downsampling experiment, 75%, 50%, or 25% of the original partial-scan points are randomly retained before fixed-size network sampling. For the pose experiment, bounded translation and rotation perturbations of 0.02 m/$1^{\circ }$, 0.05 m/$3^{\circ }$, and 0.10 m/$5^{\circ }$ are applied to the sensor poses before ray casting. The partial scans are then regenerated using the perturbed poses, while the complete target point clouds remain unchanged.

CasFusionNet, ProtoFormer, and SDD-PCN are evaluated because all three methods jointly produce completed geometry and semantic predictions. Each perturbed result is averaged over the five fixed random seeds 2025–2029 and all 276 test scenes. CD-L2 and mIoU are reported to assess geometric and semantic robustness, respectively.

As shown in Table 11, CD-L2 increases and mIoU decreases monotonically with the severity of each perturbation. Nevertheless, SDD-PCN retains the best geometric and semantic results under all nine perturbed conditions. Under the most severe range noise, random retention, and pose perturbation, the CD-L2 of SDD-PCN increases by 25.61%, 26.13%, and 19.45%, respectively, relative to the clean input, while mIoU decreases by 3.84, 5.09, and 3.47 percentage points. Thus, random retention of only 25% of the observed points produces the largest semantic degradation, whereas pose perturbation has a comparatively smaller effect. Compared with ProtoFormer under the same three severe conditions, SDD-PCN reduces CD-L2 by 10.50%, 11.33%, and 10.37%, respectively, and improves mIoU by 2.37, 2.38, and 2.33 percentage points. These results demonstrate that the proposed structure-aware representation remains beneficial under controlled measurement, density, and pose perturbations, although performance still declines as the input becomes increasingly corrupted.

#### 5.6.2. Quantitative Computational Efficiency

Table 12 compares the computational efficiency of all completion and joint completion–segmentation methods evaluated in this study. All measurements use FP32 inference on a single NVIDIA GeForce RTX 3090 GPU with a batch size of one and 16,384 input and output points. Each model is evaluated in inference mode without gradient calculation. After 50 warm-up passes, latency is averaged over 200 forward passes with CUDA synchronization before and after each measurement. Peak GPU memory is recorded using torch.cuda.max_memory_allocated. Data loading, disk input/output, and visualization are excluded.

PF-Net and SnowflakeNet have lower inference latency than SDD-PCN, but they perform geometric completion only. Among the methods that jointly produce completed geometry and semantic predictions, SDD-PCN has the lowest parameter count, peak GPU memory consumption, and inference latency. It requires 22.42 million parameters and 3.85 GB of peak memory and processes one scene in 48.6 ms, corresponding to 20.6 scenes per second.

Compared with ProtoFormer, SDD-PCN reduces the parameter count by 30.16%, peak GPU memory consumption by 28.97%, and inference latency by 37.05%. Compared with CasFusionNet, the corresponding reductions are 20.92%, 20.78%, and 29.26%. These measurements support the computational advantage of SDD-PCN among joint completion and semantic prediction methods while avoiding a direct efficiency claim against completion-only models with narrower outputs.

Beyond these quantitative measurements, SDD-PCN has an architectural task-integration advantage: its completion and semantic segmentation streams share the global scene descriptor, structure-aware branch features, resolution-expansion operations, and cross-task fused representations. A single forward pass produces both completed coordinates and semantic probabilities without requiring an independent post-completion segmentation stage. This architectural observation is presented separately from the quantitative efficiency comparison.

## 6. Discussion

### 6.1. Structure-Aware Joint Learning for Indoor Scene Completion

Indoor scenes contain two geometrically distinct components: large architectural surfaces, such as walls, floors, ceilings, beams, and columns, and comparatively small interior objects with diverse shapes and spatial arrangements. Directly encoding all points through a single undifferentiated stream can obscure this structural distinction because the dominant architectural surfaces tend to occupy substantially more points than the object instances. This observation is consistent with previous studies showing that effective point-cloud processing requires the simultaneous preservation of global spatial organization and local geometric detail [58,59,60]. The proposed structure-aware feature extraction module addresses this issue by assigning architectural and object points to dedicated encoding branches before feature fusion. The resulting representation preserves the global room layout while retaining object-level characteristics, which explains the substantial improvement from an average CD-L2 of 27.23 for the baseline network to 19.35 after introducing structure-aware feature extraction. This behavior also agrees with recent completion networks that emphasize hierarchical representations and structure-preserving point generation [61,62].

Completion and semantic segmentation are treated as complementary rather than independent objectives in the proposed framework. Geometric completion encourages the network to infer spatially coherent surfaces and object extents, whereas semantic supervision constrains the reconstructed features to remain discriminative across indoor categories. Multi-task learning studies have shown that related prediction objectives can improve shared representations when their common information is retained while task-specific decoding is properly separated [63,64]. Accordingly, the dual-decoder design shares the structure-aware latent representation but uses separate completion and semantic decoding streams. The resulting model achieves the best overall completion accuracy while maintaining an OA of 87.23%, an mAcc of 74.33%, and an mIoU of 63.62% for semantic segmentation. These results indicate that semantic supervision provides a useful regularization signal for geometric reconstruction, although the segmentation branch remains less specialized than dedicated semantic segmentation architectures [65,66].

### 6.2. Partial-Scan Simulation and Robustness to Incompleteness

The missing regions observed in real LiDAR scans are governed by sensor pose, field of view, angular sampling, surface visibility, and occlusion, rather than by independent random point removal. LiDAR studies have demonstrated that these acquisition factors produce spatially correlated gaps, uneven sampling densities, and viewpoint-dependent visibility patterns [67,68,69]. Partial-S3DIS therefore generates incomplete observations through mesh-based ray casting from multiple sensor configurations. The three pitch angles and two scanning directions used for each room produce six partial scans with different visibility patterns while retaining the original S3DIS semantic annotations. This design creates a controlled correspondence between partial observations, complete point clouds, and pointwise semantic labels, enabling completion and segmentation to be evaluated under consistent scene geometry.

The robustness results further show that the benefit of SDD-PCN increases as less of the original scene remains visible. For completion ratios between 0.40 and 0.60 and between 0.20 and 0.40, SDD-PCN achieves the lowest CD-L2 among all compared methods, indicating that its structural representation is particularly effective when large regions must be inferred. This result is consistent with completion research emphasizing the importance of hierarchical shape priors and progressive point generation under severe incompleteness [60,62,70]. For the relatively mild 0.60–0.80 interval, GRNet obtains a slightly lower CD-L2 of 16.88 than the 17.48 achieved by SDD-PCN. Therefore, the main strength of the proposed model lies not in every lightly occluded case, but in preserving global room structure and reconstructing missing objects when the observed evidence becomes substantially limited. The additional perturbation experiments extend the robustness analysis beyond the original completion-ratio stratification. SDD-PCN consistently retains lower geometric error and higher semantic accuracy than the two task-matched baselines under range noise, random point retention, and sensor-pose perturbations. Its relative advantage over ProtoFormer also remains stable or increases as the perturbations become more severe. This behavior suggests that separating architectural structures from interior objects provides a useful inductive bias when local measurements are noisy, sparse, or altered by changes in sensor pose. Nevertheless, these controlled perturbations do not eliminate the domain gap between Partial-S3DIS and physical LiDAR measurements. The experiments isolate specific acquisition deviations while retaining reconstructed meshes, synthetic ray casting, static scenes, and the original S3DIS geometry and annotations. They therefore demonstrate robustness to controlled input and acquisition perturbations but do not establish generalization to real mobile or terrestrial LiDAR scans.

### 6.3. Limitations and Future Directions

Several limitations remain. First, although the ray-casting process models viewpoint-dependent visibility and occlusion, Partial-S3DIS is constructed from reconstructed indoor meshes and does not reproduce all properties of physical LiDAR acquisition. Measurement noise, reflectance-dependent returns, mixed pixels, dynamic objects, and device-specific sampling patterns are not explicitly simulated. Consequently, performance on Partial-S3DIS does not by itself establish generalization to real mobile or terrestrial LiDAR scans. Second, the segmentation results remain constrained by class imbalance and the limited geometric evidence available for small or heavily occluded objects, which are persistent challenges in indoor point-cloud understanding [65,71,72]. Chamfer distance also evaluates geometric proximity without fully measuring surface continuity, perceptual plausibility, or the semantic correctness of newly generated regions.

Future work will therefore validate SDD-PCN on real partial scans and cross-dataset settings, including area-held-out evaluation that more strictly separates training and testing environments. The evaluation can also be expanded with F-score, Earth Mover’s Distance, missing-region accuracy, repeated-run confidence intervals, per-class completion quality, parameter count, memory consumption, and measured inference latency. On the modeling side, uncertainty-aware semantic prediction, adaptive task-loss balancing, and more explicit interaction between generated geometry and semantic features may further reduce conflicts between the two decoding objectives [60,63]. Incorporating density-aware refinement and stronger local boundary modeling is also a promising direction for improving thin structures and small indoor objects [70,71].

## 7. Conclusions

Reliable indoor scene understanding remains difficult when LiDAR observations are sparse, occluded, and unevenly distributed, because geometric recovery and semantic interpretation must be achieved from the same incomplete evidence. To address this problem, we proposed SDD-PCN, which separates architectural structures from interior objects, learns structure-specific multi-resolution features, and jointly predicts completed coordinates and semantic labels through a Dual-Decoder. We also constructed Partial-S3DIS, comprising 1,632 partial–complete scene pairs generated through sensor-aware LiDAR simulation. On this dataset, SDD-PCN achieved CD-L1 and CD-L2 values of 16.73 and 15.27, respectively. Relative to ProtoFormer, the strongest overall baseline, it reduced CD-L1 by 3.96% and CD-L2 by 7.90%, while achieving the lowest completion error in eight of ten scene categories. SDD-PCN also achieved an OA of 87.23%, an mAcc of 74.33%, and an mIoU of 63.62%, outperforming the task-matched semantic scene completion baselines across all three semantic metrics. The ablation and robustness results confirm that structure-aware encoding and cross-task feature interaction jointly improve completion quality, particularly under moderate and severe missingness. These findings demonstrate the value of explicitly modeling indoor structural heterogeneity for unified point-based completion and semantic understanding, while future work should further evaluate the framework using real-world multi-sensor scans and more diverse indoor environments.

## Figures and Tables

**Figure 1:**
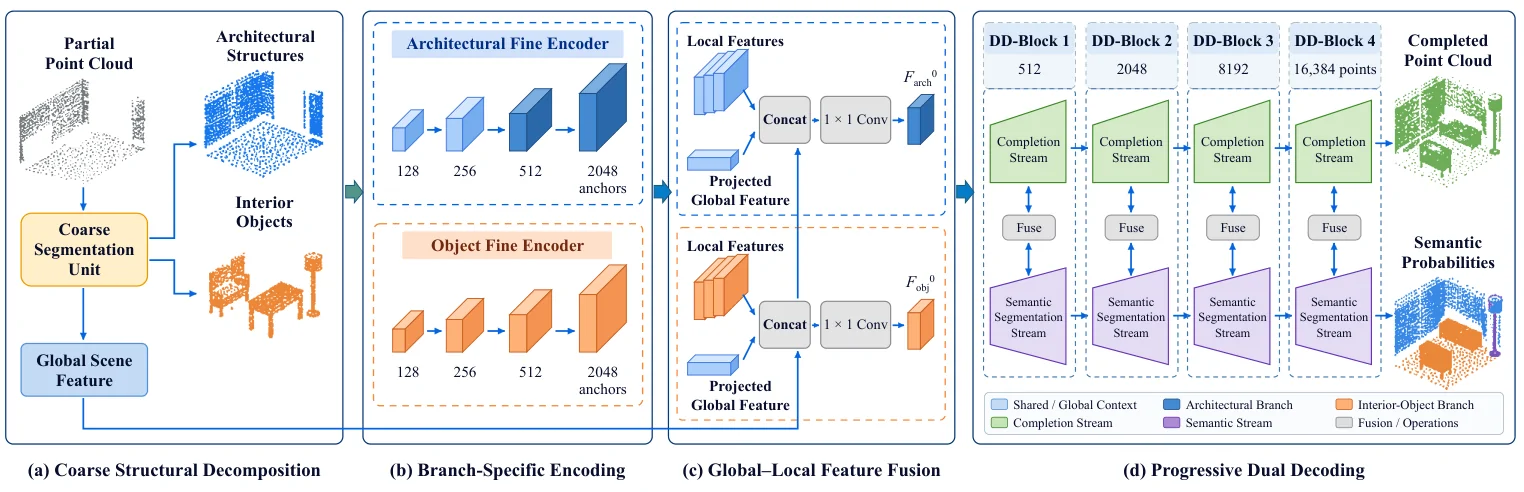
Stepwise forward pass of SDD-PCN. (**a**) The Coarse Segmentation Unit predicts binary structural labels, separates the partial input into architectural and interior-object subsets, and extracts a global scene feature. (**b**) Two independently parameterized Fine Encoders learn multi-resolution local representations from the corresponding structural subsets. (**c**) The branch-specific local features are combined with a projected and repeated global scene descriptor to obtain the fused features $F_{r}^{0}$. (**d**) Four Dual-Decoder Blocks progressively increase the output resolution from 512 to 16,384 points. Each block contains completion and semantic segmentation streams whose features are fused before the next decoding level. The final branch outputs are concatenated to produce the completed coordinates and semantic probabilities.

**Figure 2:**
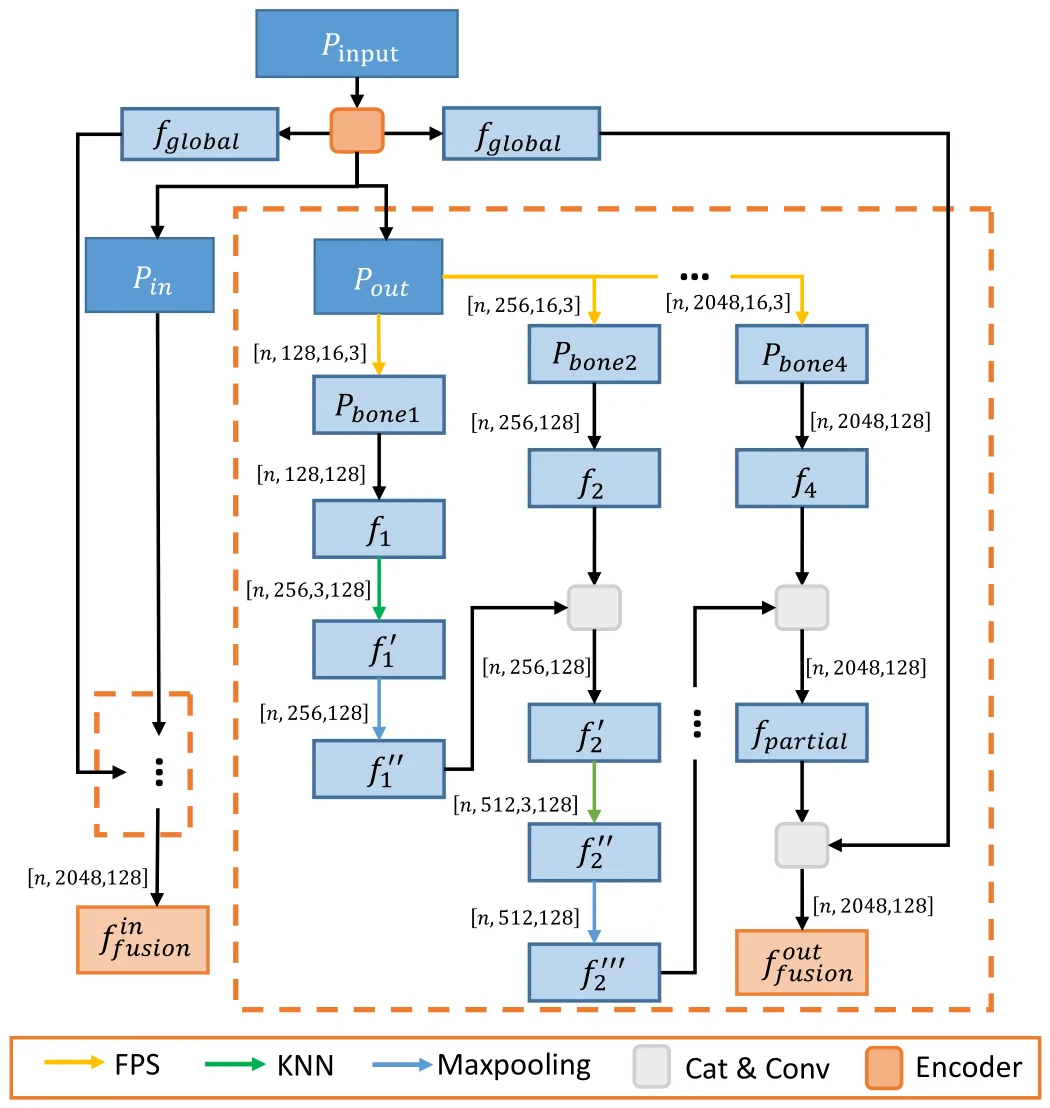
Structure-Aware Feature Extraction module. The Coarse Segmentation Unit divides the partial scene into architectural structures and interior objects while extracting a global scene feature. The Fine Encoder performs hierarchical neighborhood aggregation and cross-resolution feature propagation. The resulting local and global features are fused to produce the structure-aware representation supplied to the Dual-Decoder. The orange dashed boxes indicate the Fine Encoder branches; ellipses indicate omitted intermediate operations with the same encoder topology.

**Figure 3:**
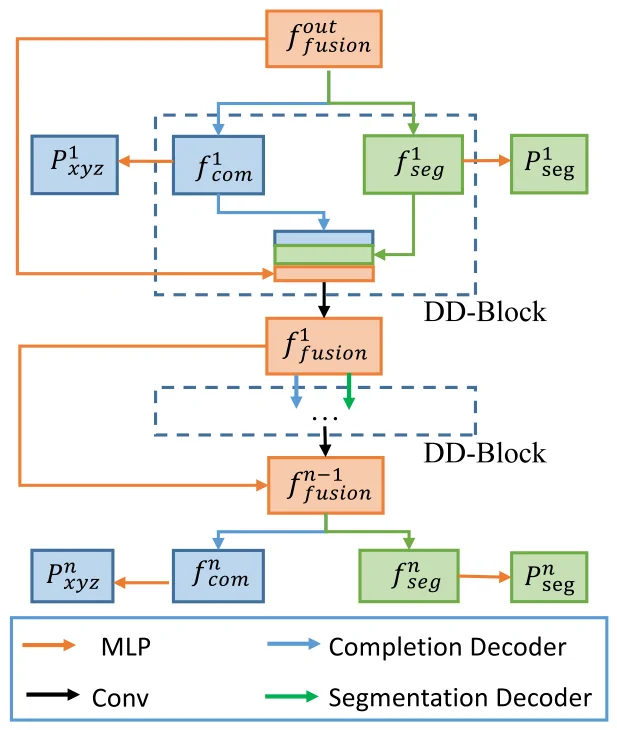
Architecture of the Dual-Decoder. Each DD-Block contains parallel completion and semantic segmentation streams. The completion stream predicts geometric features and point coordinates, whereas the segmentation stream predicts semantic features and class probabilities. Their task-specific features are fused and propagated to the next DD-Block, enabling progressive geometric and semantic refinement.

**Figure 4:**
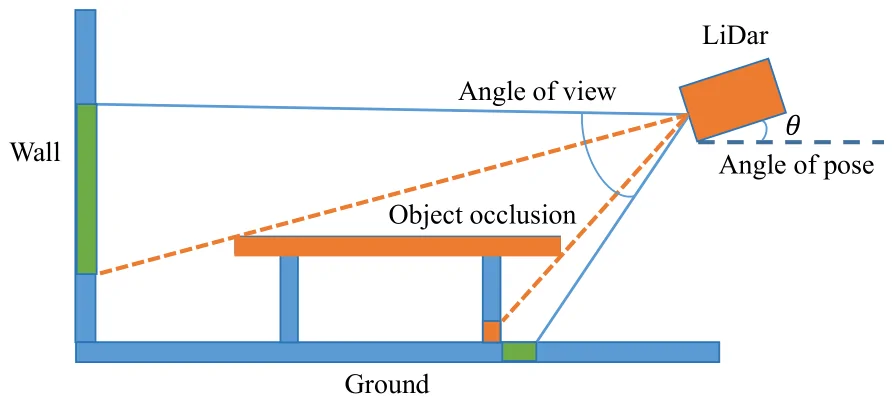
Geometric model of the indoor LiDAR simulation. The sensor field of view, mounting angle, visible surfaces, object-occlusion regions, and sensor blind regions jointly determine the observed portion of the complete scene. Green surface segments indicate visible regions. Orange dashed lines illustrate occlusion boundaries, and blue solid rays delimit the field of view.

**Figure 5:**
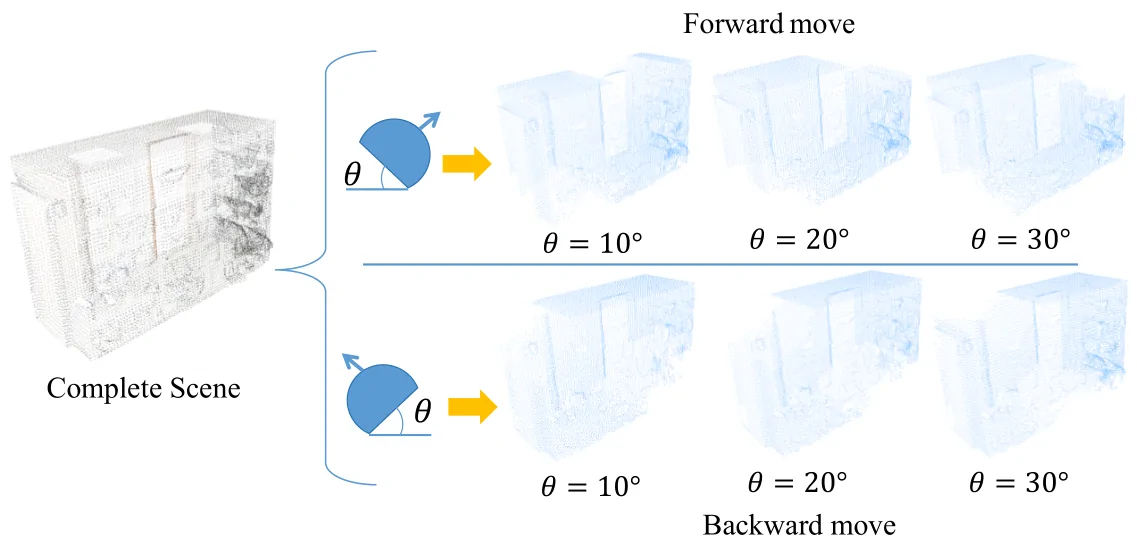
Generation of six partial scans from one complete scene. Three downward LiDAR pitch angles are combined with forward and backward platform motion. Changes in sensor orientation and viewing direction produce different blind regions and object-occlusion patterns.

**Figure 6:**
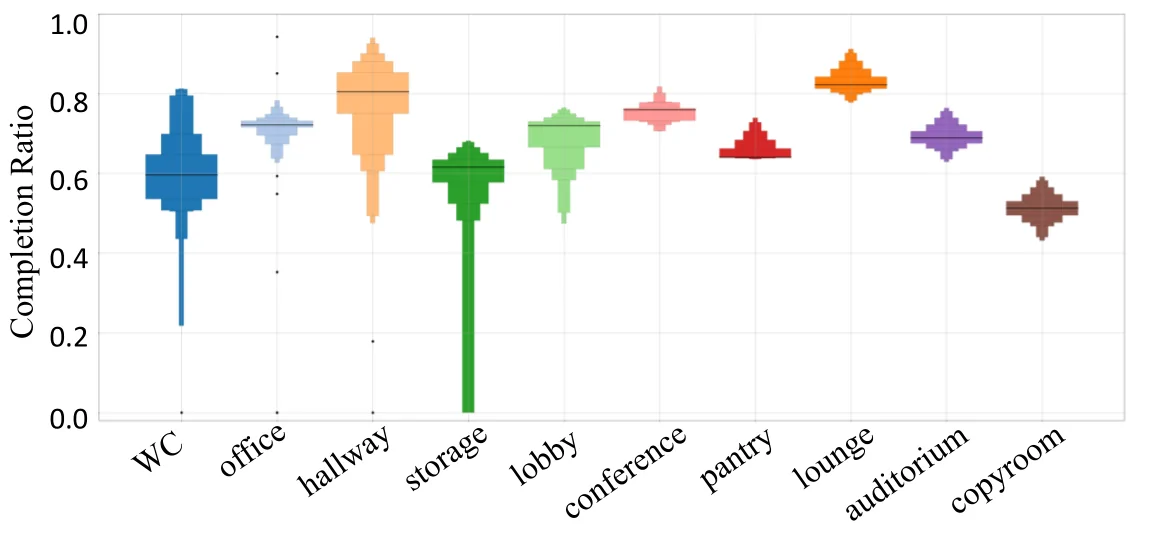
Distribution of completion ratios across the ten Partial-S3DIS scene categories. The completion ratio is defined as the number of partial-scan points divided by the number of complete-scene points. Lower ratios represent more severe incompleteness.

**Figure 7:**
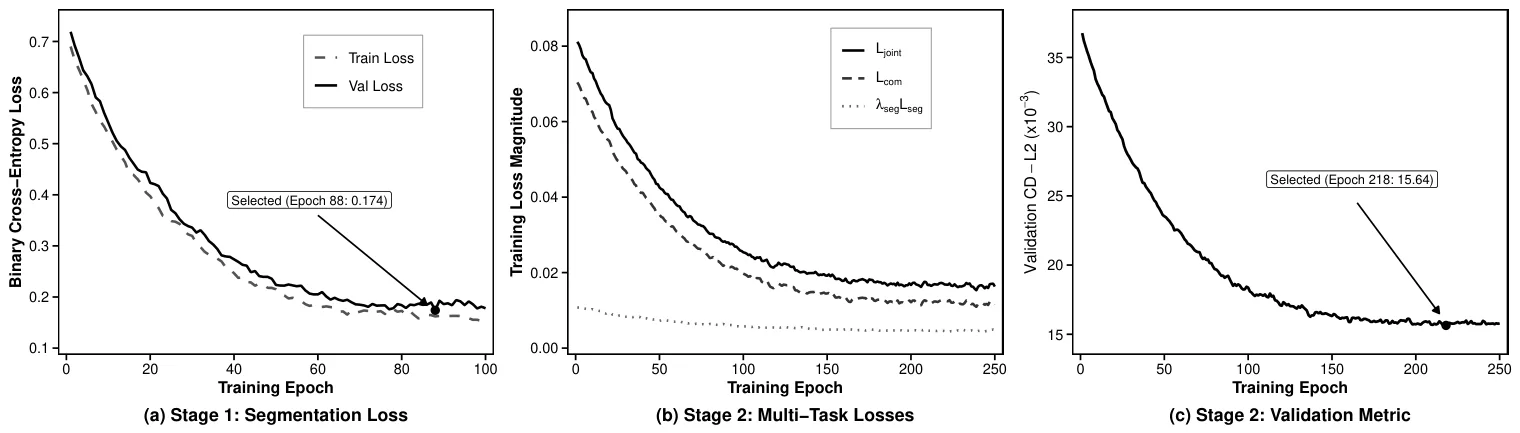
Training and validation behavior of the two-stage optimization procedure. (**a**) Training and validation binary cross-entropy losses during the 100-epoch optimization of the Coarse Segmentation Unit. (**b**) Training completion loss $L_{\mathrm{com}}$, weighted semantic loss $\lambda_{\mathrm{seg}}L_{\mathrm{seg}}$, and joint loss $L_{\mathrm{joint}}$ during the 250-epoch optimization of the Fine Encoders and Dual-Decoders. (**c**) Validation CD-L2 used for final model selection. In panel (**b**), the joint, completion, and weighted semantic losses are shown by solid, dashed, and dotted lines, respectively.

**Figure 8:**
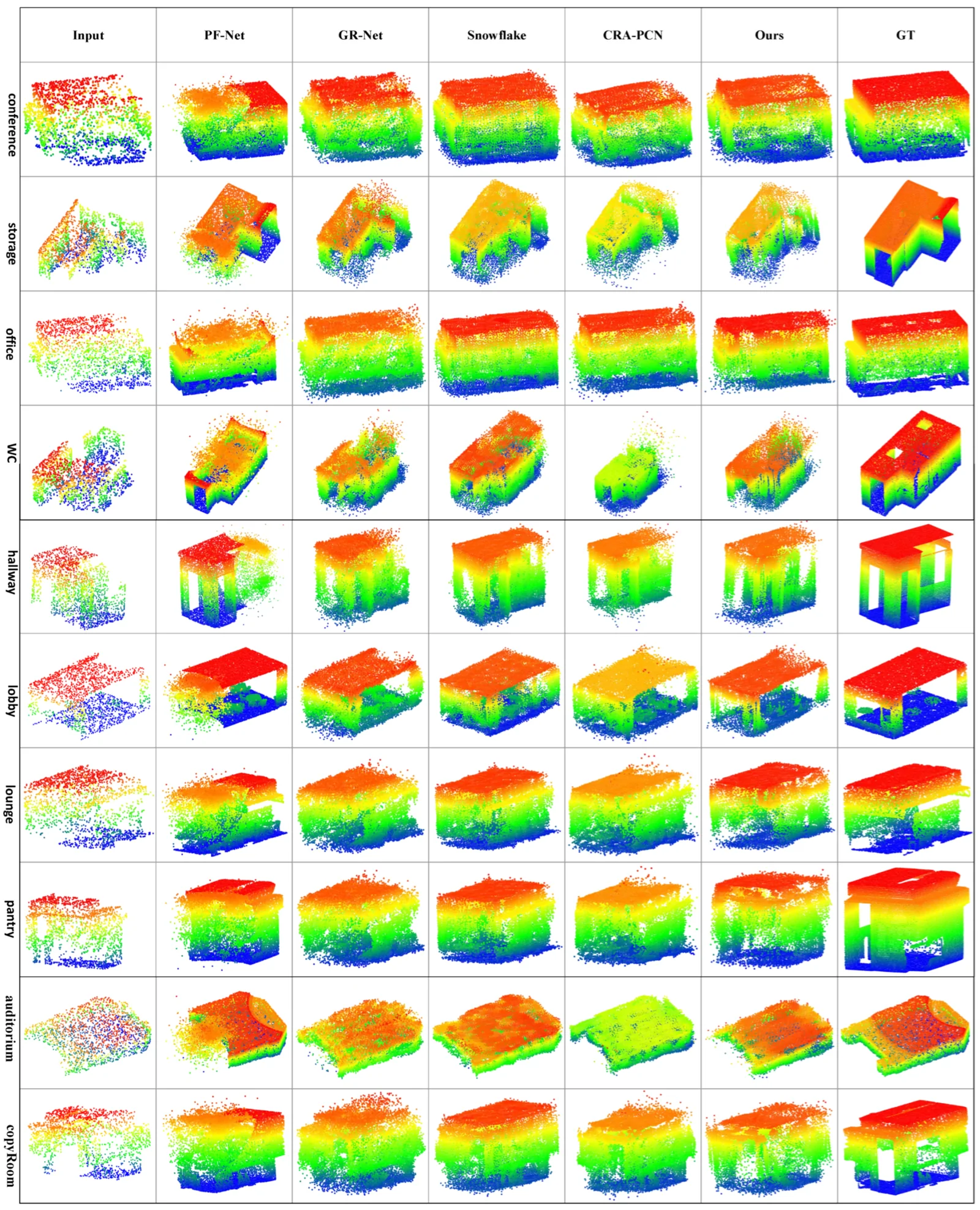
Qualitative comparison of point-cloud completion results. All methods receive the same partial input and generate 16,384 output points. Colors encode the vertical coordinate, with warmer colors representing greater height. GT denotes the corresponding ground-truth complete point cloud.

**Figure 9:**
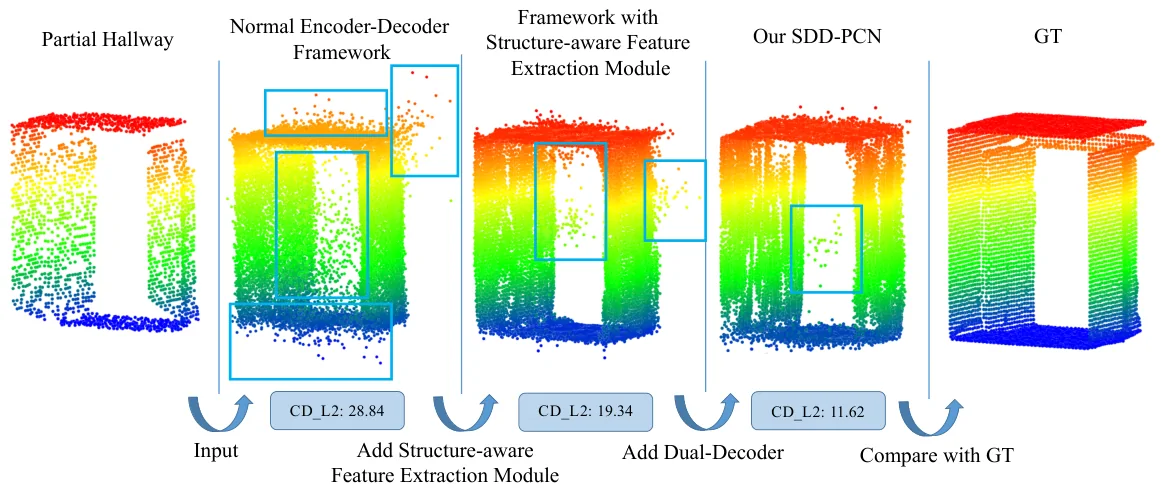
Qualitative ablation study on a hallway scene. Colors represent height, with warmer colors indicating greater height. Blue boxes highlight reconstruction errors, including outliers and incorrectly filled openings. The displayed values are CD-L2 multiplied by $10^{3}$. SFE reduces the error from 28.84 to 19.34, and the Dual-Decoder further reduces it to 11.62. GT denotes the corresponding ground-truth complete point cloud.

**Figure 10:**
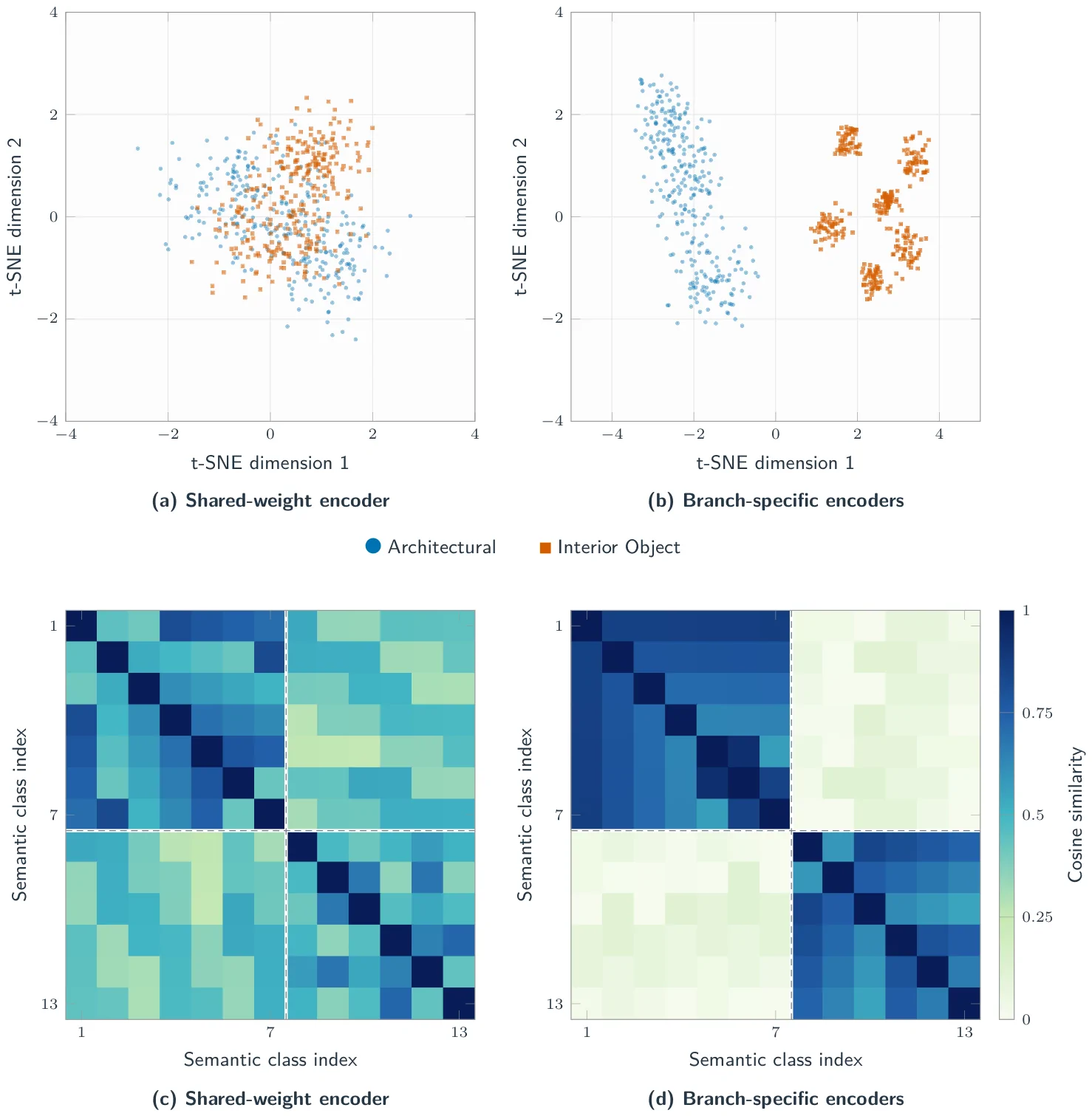
Feature-space comparison between shared-weight and branch-specific encoding. Panels (**a**,**b**) show t-SNE embeddings of the 128-dimensional fused features produced by the shared-weight encoder and branch-specific encoders, respectively. Points are distinguished according to the two coarse structural categories: architectural structures and interior objects. Panels (**c**,**d**) show the cosine-similarity matrices of the 13 semantic-class feature centroids for the corresponding encoder configurations. Class indices 1–13 represent ceiling, floor, wall, beam, column, window, door, table, chair, sofa, bookcase, board, and clutter, respectively. The dashed lines separate the seven architectural classes from the six interior-object classes. The silhouette scores were calculated from the original 128-dimensional features rather than from the two-dimensional t-SNE coordinates.

**Table 1:** Summary of the Principal Mathematical Notation.

Symbol	Definition
$P^{p}$, $P^{g}$	Partial input point set and complete ground-truth point set.
$N_{p}$, $N_{g}$	Numbers of points in $P^{p}$ and $P^{g}$, respectively.
*C*	Number of semantic classes; C=13 for Partial-S3DIS.
$C_{\mathrm{arch}}$, $C_{\mathrm{obj}}$	Architectural and interior-object semantic-class sets.
b(·), $b_{i}$, ${\hat{b}}_{i}$	Binary structural mapping, ground-truth structural label, and predicted structural label.
*r*	Structural-branch index, where r∈{arch,obj}.
*l*	Fine Encoder resolution index, where l∈{1,2,3,4}.
*j*	Dual-Decoder level index, where j∈{1,2,3,4}.
$f_{g}$	A 1024-dimensional global scene feature.
$A_{r}^{l}$	Anchor-point set of branch *r* at encoder resolution *l*.
$A_{l}$	Number of anchors at encoder resolution *l*.
$H_{r}^{l}$	Local feature matrix of branch *r* at encoder resolution *l*.
$F_{r}^{j}$	Fused feature matrix transmitted at decoder level *j*; $F_{r}^{0}$ is the initial global–local fused feature.
$M_{j}$	Total point budget at decoder level *j*.
$M_{r}^{j}$	Point budget assigned to branch *r* at decoder level *j*.
${\hat{X}}_{r}^{j}$	Predicted coordinate matrix for branch *r* at decoder level *j*.
$Z_{r}^{j}$	Semantic-logit matrix for branch *r* at decoder level *j*.
${\hat{P}}_{r}^{j}$	Semantic-probability matrix for branch *r* at decoder level *j*.
${\hat{P}}_{r}^{j}$	Predicted point set for branch *r* at decoder level *j*.
$\alpha_{j}$, $\beta_{j}$	Completion- and semantic-loss weights at decoder level *j*.
$\lambda_{\mathrm{seg}}$	Coefficient controlling the contribution of semantic loss to the joint objective.

**Table 2:** Room-Level Distribution of Partial-S3DIS.

Scene Category	Training	Validation	Test	Total
Conference room	7	2	2	11
Storage room	14	3	2	19
Office	106	25	25	156
WC	5	3	3	11
Lobby	1	1	1	3
Lounge	1	1	1	3
Auditorium	1	1	1	3
Copy room	1	1	1	3
Hallway	42	9	9	60
Pantry	1	1	1	3
Total	179	47	46	272

**Table 3:** Overall Geometric Completion Performance on Partial-S3DIS.

Method	CD-L1 $\times 10^{3}$ ↓	CD-L2 $\times 10^{3}$ ↓
PF-Net	26.11	23.57
GRNet	20.35	20.16
SnowflakeNet	23.12	19.96
CRA-PCN	19.21	18.95
CasFusionNet	18.84	18.27
ProtoFormer	17.42	16.58
SDD-PCN	**16.73**	**15.27**

Note: ↓ indicates that lower values are better. Bold values indicate the best results among the compared methods. Overall results are strict pair-level averages over the 276 test scenes.

**Table 4:** Category-Level CD-L2 Results on Partial-S3DIS ($\times 10^{3}$).

Method	Conference	Storage	Office	WC	Hallway	Auditorium	Copy Room	Lobby	Lounge	Pantry
PF-Net	28.86	28.13	24.43	22.31	18.74	34.23	29.12	16.29	14.24	29.56
GRNet	23.73	32.53	20.34	23.55	15.41	16.96	29.41	**12.07**	**12.97**	25.69
SnowflakeNet	19.83	34.30	20.05	18.75	16.00	18.03	25.84	17.00	22.51	24.99
CRA-PCN	18.99	22.72	19.73	19.64	16.11	16.13	20.92	15.37	18.39	20.24
CasFusionNet	18.16	22.04	19.04	18.63	15.38	16.27	21.19	14.82	17.55	19.78
ProtoFormer	16.62	19.48	17.75	16.35	12.91	14.81	19.95	13.64	15.29	17.83
SDD-PCN	**15.24**	**17.56**	**16.80**	**14.74**	**10.75**	**13.57**	**18.71**	12.43	14.62	**16.46**

Bold values indicate the best results among the compared methods.

**Table 5:** Structure-Specific Geometric Evaluation on Partial-S3DIS.

Method	Ceiling PFE (cm) ↓	Floor PFE (cm) ↓	Wall PFE (cm) ↓	Mean PFE (cm) ↓	Door FFR (%) ↓	Window FFR (%) ↓
PF-Net	3.82	3.15	4.13	3.70	31.2	28.5
GRNet	2.76	2.48	3.25	2.83	24.1	22.7
SnowflakeNet	3.14	2.86	3.61	3.20	35.6	32.1
CRA-PCN	2.41	2.12	2.87	2.47	20.3	18.6
CasFusionNet	2.33	2.17	2.74	2.41	18.5	17.2
ProtoFormer	1.98	**1.83**	2.45	2.09	16.4	**14.7**
SDD-PCN	**1.62**	1.85	**1.93**	**1.80**	**12.3**	15.2

Note: ↓ indicates that lower values are better. PFE is reported in centimeters. Mean PFE is the macro-average across the ceiling, floor, and wall categories. FFR is reported as a percentage. Bold values indicate the best result in each column.

**Table 6:** Comparison with Point-Based Semantic Scene Completion Methods on Partial-S3DIS.

Method	CD-L1 $\times 10^{3}$ ↓	CD-L2 $\times 10^{3}$ ↓	OA (%) ↑	mAcc (%) ↑	mIoU (%) ↑
CasFusionNet	18.84	18.27	85.16	71.48	59.25
ProtoFormer	17.42	16.58	86.71	73.65	62.18
SDD-PCN	**16.73**	**15.27**	**87.23**	**74.33**	**63.62**

↓ indicates lower is better; ↑ indicates higher is better. Bold values indicate the best results among the compared methods.

**Table 7:** Semantic Segmentation Results on Completed Partial-S3DIS Scenes.

Method	OA (%)	mAcc (%)	mIoU (%)
PointNet [44]	78.53	66.27	47.60
PointCNN [54]	**88.15**	75.64	65.48
SPGraph [55]	85.57	73.02	62.14
PointWeb [56]	87.30	**76.22**	66.74
ShellNet [57]	87.14	76.03	**66.82**
SDD-PCN	87.23	74.33	63.62

Bold values indicate the best results among the compared methods.

**Table 8:** Per-Class IoU on the Partial-S3DIS Test Set (%).

Method	Ceiling	Floor	Wall	Beam	Column	Window	Door	Table	Chair	Sofa	Bookcase	Board	Clutter	mIoU
CasFusionNet	88.42	91.56	75.83	40.17	42.64	48.21	52.38	67.15	72.84	45.39	51.72	46.85	**47.09**	59.25
ProtoFormer	90.15	**94.27**	78.46	43.82	45.19	52.74	56.81	69.35	75.12	**51.68**	55.43	49.21	46.11	62.18
SDD-PCN	**92.53**	93.81	**79.84**	**47.21**	**46.38**	**53.64**	**59.72**	**69.85**	**76.14**	50.42	**58.62**	**52.32**	46.58	**63.62**

Note: The mIoU in the final column is the arithmetic mean of the 13 class-specific IoU values. Bold values indicate the best result in each column.

**Table 9:** Ablation Study of Structure-Aware Feature Extraction and the Dual-Decoder.

Configuration	Overall	Conference	Storage	Office	WC	Hallway	Auditorium	Copy Room	Lobby	Lounge	Pantry
Standard encoder–decoder	27.94	25.99	28.72	27.73	30.94	28.84	24.13	31.96	19.30	26.69	28.04
With SFE	19.01	18.31	21.61	18.37	22.71	19.34	16.29	20.11	16.27	18.91	21.54
SFE + Dual-Decoder	**15.27**	**15.24**	**17.56**	**16.80**	**14.74**	**10.75**	**13.57**	**18.71**	**12.43**	**14.62**	**16.46**

Note: Bold values indicate the best results among the compared methods. CD-L2 values are reported under the $\times 10^{3}$ convention. The Overall column reports strict pair-level averages over the 276 test scenes rather than unweighted averages across the ten scene categories. Lower values indicate better completion performance.

**Table 10:** Ablation Study of Shared versus Branch-Specific Encoding on Partial-S3DIS.

Configuration	CD-L1 ($\times 10^{-3}$) ↓	CD-L2 ($\times 10^{-3}$) ↓	OA (%) ↑	mAcc (%) ↑	mIoU (%) ↑
Unified shared encoder + Dual-Decoder	21.14	19.63	82.17	68.52	53.84
Partitioned shared-weight encoder + Dual-Decoder	18.67	17.39	85.21	71.86	59.75
Branch-specific encoders + Dual-Decoder	**16.73**	**15.27**	**87.23**	**74.33**	**63.62**

↓ indicates lower is better; ↑ indicates higher is better. Bold values indicate the best results among the compared methods.

**Table 11:** Robustness to Input and Acquisition Perturbations on Partial-S3DIS.

Perturbation	Level	CasFusionNet		ProtoFormer		SDD-PCN	
		CD-L2↓	mIoU↑	CD-L2↓	mIoU↑	CD-L2↓	mIoU↑
None	Clean	18.27	59.25	16.58	62.18	**15.27**	**63.62**
Range noise	0.005 m	18.94	58.64	17.21	61.59	**15.82**	**63.14**
	0.010 m	20.42	57.18	18.67	60.23	**17.06**	**62.05**
	0.020 m	23.65	54.32	21.43	57.41	**19.18**	**59.78**
Random retention	75%	19.06	58.37	17.34	61.42	**15.79**	**62.97**
	50%	20.73	56.41	18.86	59.68	**16.94**	**61.46**
	25%	23.95	52.86	21.72	56.15	**19.26**	**58.53**
Pose perturbation	0.02 m/$1^{\circ }$	18.79	58.71	17.07	61.66	**15.68**	**63.19**
	0.05 m/$3^{\circ }$	20.08	57.04	18.29	60.18	**16.63**	**62.01**
	0.10 m/$5^{\circ }$	22.46	54.63	20.35	57.82	**18.24**	**60.15**

Note: ↓ indicates lower is better; ↑ indicates higher is better. Bold values indicate the best result for each metric at each perturbation level among the compared methods. Range-noise levels denote the standard deviation of zero-mean Gaussian noise added along the LiDAR ray direction. Random retention is applied to each original partial scan before fixed-size network sampling. Pose-perturbation levels denote the maximum absolute translation and rotation perturbations applied to each sensor pose before ray casting. Each perturbed result is the mean over five fixed random seeds (2025–2029) and all 276 test scenes. CD-L2 is multiplied by $10^{3}$, and mIoU is reported as a percentage.

**Table 12:** Comparative Computational Efficiency on Partial-S3DIS.

Method	Output Tasks	Parameters (M) ↓	Peak Memory (GB) ↓	Latency (ms/Scene) ↓	Throughput (Scenes/s) ↑
PF-Net	Completion	2.45	1.62	22.4	44.6
GRNet	Completion	76.50	6.95	64.8	15.4
SnowflakeNet	Completion	19.24	3.48	41.5	24.1
CRA-PCN	Completion	24.68	4.25	56.3	17.8
CasFusionNet	Completion + semantics	28.35	4.86	68.7	14.6
ProtoFormer	Completion + semantics	32.10	5.42	77.2	13.0
SDD-PCN	Completion + semantics	22.42	3.85	48.6	20.6

Note: ↓ indicates lower is better; ↑ indicates higher is better. Measurements were obtained on a single NVIDIA GeForce RTX 3090 GPU with a batch size of one and 16,384 input and output points. Throughput is calculated as 1000/latency. Completion-only methods do not generate semantic predictions.

## Data Availability

The original contributions presented in this study are included in the article. Further inquiries can be directed to the corresponding author.
